# Transcriptional profiles of the fish parasite *Neoechinorhynchus agilis* (Acanthocephala) emphasize energetic stress in males and high cell-division activity in females

**DOI:** 10.1186/s12864-025-12298-y

**Published:** 2025-12-09

**Authors:** Camille-Sophie Cozzarolo, Alexandros Vasilikopoulos, Olivier De Thier, Laura Hagemann, Bahram Sayyaf Dezfuli, Karine van Doninck, Holger Herlyn

**Affiliations:** 1 Institute of Organismic and Molecular Evolution, Johannes Gutenberg University Mainz, Anselm-Franz-von-Bentzel-Weg 7, 55128 Mainz, Germany; 2 Research Unit of Molecular Biology and Evolution, Université Libre de Bruxelles (ULB), Avenue F. D. Roosevelt 50, Brussels, 1050 Belgium; 3 Centre for Human Genetics, University Hospitals Leuven (UZ Leuven), Herestraat 49, Leuven, 3000 Belgium; 4 Department of Life Sciences & Biotechnology, University of Ferrara, St. Borsari 46, Ferrara, 44121 Italy

**Keywords:** Genomics, Transcriptomics, Sex-biased gene expression, Energy, Fertility, Animal production, Parasite control, Disease

## Abstract

**Background:**

Thorny-headed worms (Acanthocephala) occur worldwide in gnathostome vertebrates feeding on mandibulate arthropods. They can manipulate host behavior, accumulate heavy metals, and have lately gained economic relevance as a pest in fish aquaculture. Yet, despite their ecological and economic significance, little is known about the gene-expressional background of acanthocephalan development, maturation, and reproduction in the definitive host. To fill this gap in knowledge, we studied *Neoechinorhynchus agilis* (Eoacanthocephala) specimens sampled from the digestive tracts of naturally infected thin-lipped mullets (*Chelon ramada*).

**Results:**

We generated a nuclear draft genome and a whole-body transcriptome assembly. Differential expression analysis based on transcript abundances of 36 males and 30 females revealed that 30% of the transcripts had sex-biased expression. Gene ontologies relating to energy metabolism and microtubules were enriched with male-biased genes; female-biased genes indicated increased cell division and transcription activity. Only 0.19% of genes were differentially expressed as a function of female size (using whole-body RNA weight as a proxy for size), versus 5.4% in males.

**Conclusions:**

Transcriptome annotations underlined energy metabolism and reproduction as major tasks in *N. agilis* life. Our results suggest that males, smaller than females and thus supposedly less competitive, struggle for sufficient energy to produce large quantities of sperm. Female-biased genes were consistent with the production and development of numerous eggs. Finally, we identified genes with particular importance in the growth or reproduction of *N. agilis*, that could be investigated as candidate targets for acanthocephalan control in fish aquaculture.

**Supplementary Information:**

The online version contains supplementary material available at 10.1186/s12864-025-12298-y.

## Introduction

From the middle of the 20th century, it became increasingly clear that Acanthocephala (thorny-headed worms) could be closely related to Rotifera [1]. Ultrastructural and molecular analyses have since substantiated that acanthocephalans are highly derived rotifers. In fact, quite many peculiarities distinguish acanthocephalans from other members of the clade, be it called Rotifera-Acanthocephala, Rotifera, or Syndermata [2–6]. For example, acanthocephalans lack an alimentary tract, take up nutrients via the body surface, and possess an eversible attachment organ armed with spines or hooks at the anterior body pole (proboscis). These and other features reflect the specific lifestyle of acanthocephalans, which are all endoparasites with a minimum of two hosts. Together with their hosts, acanthocephalans have successfully colonized terrestrial, aquatic, and aerial environments across the globe [7]–[8], whereby the intermediate host is recruited from mandibulate arthropods and definitive hosts from gnathostome vertebrates [9–11].

Human infections were more common in the distant past [9, 12] and still occur, mostly in children who might have taken up an infected cockroach [13]. Acanthocephaliasis has also been reported in farm animals such as chickens, peccaries, and domestic pigs [14–17]. In fish farming, however, thorny-headed worms have gained economic relevance [18]. Acanthocephaliasis is considered the major obstacle in the establishment of fish aquaculture in inland South America. Here, up to 100% of tambaquis (*Colossoma macropum*) are infected with hundreds of acanthocephalans (*Neoechinorhynchus buttnerae*, Eoacanthocephala) on average [19–23]. The worms damage the intestinal wall through the anchoring of their proboscis [24]–[25]. This can result in bleeding, inflammatory reactions, and necrosis [26–28]. They also deprive the fish of organic and inorganic nutrients [29–31], possibly leading to reduced growth. In addition, infected fish show cachexia and malformations, which reduce marketability [21, 32]. Control by oral administration of established anthelmintics [33] is problematic due to their low specificity [34]–[35] and general pro-apoptotic efficiency [36–38]. Thus, there is a need for the development of alternative, sustainable agents for the control of acanthocephalan pests in fish aquaculture [33, 39].

Acanthocephalans infect their mandibulate intermediate host by oral uptake of eggs. The larval stage, acanthor, hatches inside the alimentary canal, penetrates the intestinal wall, and develops in the hemocoel into a so-called acanthella around which a cyst may form [40]– [41]. If the intermediate host carrying one or several encysted acanthocephalans (cystacanths) falls prey to a suitable gnathostome vertebrate, host transfer can take place [9]– [10, 42]– [43]. There is even evidence that acanthocephalans can manipulate the behavior of the intermediate host, thus increasing the chances of infecting a definitive host [44]. Within the definitive host’s intestines, the worm continues to grow and eventually matures. Sexual maturation implies the full differentiation of male or female phenotypes, which primarily affects the internal organization of the trunk (metasoma), while there appears to be no reorganization of proboscis structures [20, 41, 45]. In females, the metasomal changes include the proliferation of fragmented ovaria (ovarian balls) that freely float in the fluid-filled body cavity, while in males, the usually paired testicles increase in size [10, 45]– [46]. Upon mating, females start producing thousands of eggs per patent period [47]– [48]. Mature eggs are then shed into the digestive tract of the definitive host, from where they are eventually released into the environment along with the feces [49]– [50].

We investigated the molecular basis of growth, sexual maturation, and reproduction in a close phylogenetic relative of *N. buttnerae*, the congener *Neoechinorhynchus agilis*, sampled from Adriatic thin-lipped mullets (*Chelon ramada*). First, we reconstructed a draft genome assembly from the DNA of a pool of male and female worms, and a transcriptome from the RNAs of 74 male and female specimens of different sizes, individually sequenced. For differential expression analysis (DEA), RNA-Seq reads were mapped to the transcriptome assembly. We then tested for sex-biased gene and size-associated gene expression, using whole-body RNA weight as a proxy for worm size. This approach allowed the identification of both shared and divergent transcriptional profiles across developing or reproducing stages in male and female worms. Gene ontology enrichment analyses functionally contextualized our findings.

## Materials and methods

### Samples

Specimens of *Neoechinorhynchus agilis* (Rudolphi, 1819) van Cleave, 1916 were retrieved from the intestines of thin-lipped mullets (*Chelon ramada*), caught in Comacchio lagoons, Italy, in December 2020 (for ONT Sequencing) and December 2022 (for mRNA-Seq). Examination of the sacrificed fish was conducted in the laboratories at the University of Ferrara. Abdomens and intestines were opened with scissors, and acanthocephalans were gathered with tweezers. The worms were examined under the stereomicroscope to identify their sex. To ensure the integrity of the animals, we did not squeeze out the bursa copulatrix of the males or the eggs of the females. This was essential to avoid RNA loss due to leakage of body fluids. Furthermore, visual inspection had to be carried out quickly to prevent changes in the transcription profile and RNA degradation. Thus, sexing mainly relied on external observation, including the subterminal genital pore of males vs. the terminal genital pore in females [51].

### Sequencing and *de novo* genome assembly

Genomic DNA was extracted from a pool of approximately 50 male and female worms using Qiagen’s DNeasy Blood & Tissue Kit. Extracted DNA was sent to Genoscope (Évry, France), where libraries were prepared for Illumina PE150 and Oxford Nanopore sequencing, respectively. Sequencing of short (Illumina) and long (Oxford Nanopore, ONT) reads was performed on an Illumina NovaSeq 6000 and a PromethION (R9.4) system. To supplement the initial Illumina PE150 data from a pool of individuals, additional reads were generated from a single female worm (female A) preserved in RNAlater. 9.3 µg of DNA was extracted using Qiagen’s Puregene Kit and sent to the Genomics Core Facility (UZ Leuven, Leuven, Belgium), where library preparation was performed using the KAPA HyperPrep PCR-free Kit and sequencing was done on an Illumina NovaSeq 6000 platform. Additional long reads were obtained by sequencing three PacBio HiFi libraries; two generated from the same extraction of this single female A, and an additional third PacBio HiFi library from the DNA extraction of a second female individual (female B) that was also preserved in RNAlater. For the second individual, 8.3 µg of DNA were extracted using Qiagen’s Puregene Kit. Standard whole-genome library preparation was performed at the Genomics Core facility, where subsequent sequencing was performed on a PacBio Sequel IIe SMRT system. Hi-C reads were generated from the tissue of yet another single female (female C) preserved in RNAlater. After fixation in formaldehyde, we followed the Arima HiC + kit (2 restriction enzymes), and the crosslinked and purified DNA was sent to Genoscope for library preparation and sequencing, yielding Illumina PE150 reads. A summary of all sequencing reads used in this study is provided in Table 1.

**Table 1:** Summary of the different reads used for the genome assembly and the total number of base pairs generated

Technology	DNA origin	Total size (Gbp)
Illumina (PE 150): 2 libraries	pool of 50 males and females single female A	17.157
Oxford Nanopore (ONT)	pool of 50 males and females	4.168
PacBio HiFi: 3 libraries	single female A twice single female B	0.088
Hi-C (Illumina PE150)	single female C	42.530

Before assembly, the two Illumina libraries were combined into one set of paired-end reads and were pre-processed using fastp v. 0.23.2 [52] with the parameters -5 20 -3 20 --detect_adapter_for_pe --trim_poly_g. Read quality was assessed using FastQC 0.11.9 for the Illumina reads [53]. We used the software Filtlong v. 0.2.0 [54] to filter ONT reads based on their quality and minimum length by also providing the Illumina reads as a reference, retaining those long reads with a minimum length of 5000 bp (options --min_length 5000 --keep_percent 90 --split 1000) [55]. The pre-processed ONT reads were then concatenated with the HiFi reads into a single “long-read” library, as the yield of PacBio reads was very low and insufficient as a standalone for *de novo* genome assembly. Subsequently, short and long reads were assembled into a contig-level assembly using MaSuRCA v. 4.0.9 [56] in hybrid mode with the parameters “PE = pe 500 50; GRAPH_KMER_SIZE = auto; USE_LINKING_MATES = 0; LIMIT_JUMP_COVERAGE = 300; CA_PARAMETERS = cgwErrorRate = 0.15; JF_SIZE = 900000000; FLYE_ASSEMBLY = 1”. Long reads were mapped to the assembly using minimap2 v. 2.27-r1193 [57] with the parameter -x map-ont, after which purge_dups v. 1.2.5 [58] was used to remove uncollapsed haplotypes [59]. We compared this genome assembly with one more genome assembly strategy that was based only on long reads, specifically ONT reads, for which we had a higher sequencing yield (see supplementary methods and Table 1). This long-read genome assembly was generated using Flye v. 2.9.1 and subsequently was polished with short reads to improve its quality [59]– [60] (see supplementary methods). Since the genome assembly that resulted from the MaSuRCA assembler displayed higher k-mer and BUSCO completeness, we decided to proceed with this genome assembly for genome scaffolding (supplementary Figs. S1 and S2, Table S1).

The selected contig-level genome assembly was scaffolded using Hi-C reads. Initially, hicstuff v. 2.1.2 [61] was applied to map the Hi-C reads on the contig-level genome assembly. Hicstuff was configured to use bowtie2 v. 2.3.4.2 [62] with the parameters -e DpnII, HinfI -m iterative (supplementary Fig. S3). Next, instaGRAAL v. 0.1.6 [63] was applied to scaffold the genome with the parameters “-l 5 -c 50”, followed by the instagraal-polish module with parameters -m polishing -j NNNNNNNNNN. Manual curation of the scaffolded assembly was performed using PretextMap v. 0.1.9 and PretextView v. 1.0.0 [64]– [65] before attempting to close gaps in the *N. agilis* scaffolds using TGS-GapCloser v. 1.0.3 [66]. Finally, scaffolds shorter than 5 kb were removed, and putative contaminant sequences were identified and manually removed using BlobTools2 v. 2.6.4 [67], based on atypical GC content, read coverage, and taxonomic classification. To achieve this, scaffolds were aligned to the UniProt reference proteome database (accessed on January 25, 2022) using DIAMOND v. 2.0.15 [68], and coverage was estimated by mapping long reads to the assembly with minimap2. The completeness of the reference genome was assessed using universal single-copy orthologs (BUSCO) v.5.4.3 against the eukaryota_odb10 and metazoa_odb10 databases [69] and with assembly spectra copy number plots generated with the K-mer analysis toolkit v. 2.4.2 [70] (supplementary Fig. S1 and S2).

### mRNA-Seq and *de novo* transcriptome assembly

RNA was isolated from single whole male and female worms (*N* = 74) with the Maxwell^®^ RSC simplyRNA Tissue Kit. Pelleted RNA was resolved in HPLC-grade H_2_O, upon which we checked RNA quality (Bioanalyzer) and concentration (Qubit). Following poly-A capture, libraries were constructed with the NEBNext Ultra II Directional RNA library preparation kit. mRNA-Seq was then conducted on an Illumina NextSeq 2000 platform, generating 25 mio paired-end reads per sample (2 × 12.5 mio reads, 2 × 150 nt). The mRNA-Seq datasets are available at NCBI GenBank under BioProject ID PRJNA1223661.

Libraries were aligned to the smr_v4.3_default_db rRNA database using SortMeRNA v. 4.3.6 [71] to filter out potential rRNA contaminants. We removed adapters (ILLUMINACLIP: TruSeq3-PE. fa:5:30:10) and low-quality fragments (SLIDINGWINDOW:5:5, MINLEN:50) with Trimmomatic v. 0.36 [72]. We then applied Rcorrector v. 1.0.4 [73] to correct random sequencing errors. Between all pre-processing steps, read quality was checked with FastQC v. 0.11.9 [53]. Pre-processed libraries were concatenated and mapped to the draft genome assembly using GSNAP v. 2021-05-27 [74] to use the genome-guided option of Trinity v. 2.11 [75] to assemble a transcriptome. The resulting assembly was evaluated with TransRate v. 1.0.3 [76]. We selected the transcripts considered as good where both members of a read pair were (1) aligned, (2) in the correct orientation, (3) on the same contig, and (4) without overlapping either end of the contig. We evaluated the transcriptome completeness using BUSCO v. 5.4.3 [69] against the eukaryota_odb10 and metazoa_odb10 databases. We also assembled individual transcriptomes for each worm using Trinity’s default mode and ran BUSCO on all these assemblies to evaluate whether core orthologs were consistently missing across the individuals. Finally, we ran the same BUSCO analysis on the transcriptome assemblies of *Pomphorhynchus laevis* (Zoega in Müller, 1776) Porta, 1908 (Acanthocephala: Palaeacanthocephala) [77] and *Seison nebaliae* Grube, 1861 (Pararotatoria: Seisonidea) [78], to assess the proportions of missing orthologs shared among these Rotifera.

### Transcriptome functional annotation

The *de novo* assembled transcriptome was scanned for open reading frames (ORF) using TransDecoder v. 5.7 [79]. We compared the longest ORFs to the PFAM database [80] using hmmscan in HMMER v. 3.3.2 [81] and performed homology transfer from the SwissProt database [82] using BLAST+ [83] to inform protein prediction with TransDecoder, using the --retain_pfam_hits and --retain_blastp_hits options. We functionally annotated the predicted proteins using the eggNOG-mapper v. 2.1.8 (transfer of functional information from orthologs; [84]– [85]) and InterProScan v. 5.70–102 (classification into families, domain and site prediction; [86]– [87]). We also ran DIAMOND v. 2.0.13.151 [68] against the Swiss-Prot and TrEMBL databases to identify homologs [82].

### Genome structural annotation

Repeats in the genome were masked by RepeatMasker v. 4.1.2 [88]. We used BRAKER v. 2.1.5, which relies on GeneMark-ET, AUGUSTUS, Bamtools, SAMtools, and DIAMOND [68, 83, 89–94] to structurally annotate the genes of the newly assembled genome based on mRNA-Seq reads. Descriptive metrics were computed with QUAST v. 5.2 [95] and AGAT [96].

### Exploration of gene expression profiles

We removed potential rRNA contaminants from raw reads using SortMeRNA v. 4.3.6 [71], as well as adapters and low-quality reads with Trimmomatic v. 0.36 (ILLUMINACLIP:TruSeq3-PE.fa:5:30:10; SLIDINGWINDOW:5:20, MINLEN:50; [72]). Transcript abundance was quantified with Salmon [76] via “quasi-mapping” the reads on the *de novo* transcriptome assembly, implementing the --seqBias, --posBias, and --gcBias options and 30 numGibbsSamples. The rest of the statistical analyses and visualization were conducted in R v. 4.4.0 [97] with extensive use of the “tidyverse” packages [98]. We imported the counts from Salmon quantification with “tximport” [99] and summarized count values for alternative transcripts (isoforms) by their genes as defined by Trinity for the transcriptome assembly. We relied on the package DESeq2 [100] for data transformation and DEA. For visualization purposes, we transformed the count data using the function “varianceStabilizingTransformation”, which removes the dependence of the variance on the mean. To explore the existence of clusters of expression profiles, we plotted a heatmap (“pheatmap”; [101]) with the variance-stabilized counts of the 100 genes exhibiting the highest expression levels, and conducted a Principal Component Analysis (PCA) with the variance-stabilized counts of all the genes. These preliminary analyses highlighted several specimens that did not cluster according to the visually assigned sex (supplementary Figs. S4 and S5). We computed a sample-to-sample distance matrix based on the expression of genes homologous or orthologous to genes found to be sex-biased in the palaeacanthocephalan *P. laevis* [102]. Then, we conducted hierarchical clustering to determine clusters of same-sex individuals (see supplementary methods) and eliminated the mis-sexed individuals.

### Differential expression analysis

Intending to identify genes involved in the late development and reproduction of *N. agilis*, we used worms’ whole-body RNA content as a proxy for their size and, indirectly, for their age. As acanthocephalans show sexual size dimorphism, we scaled the individual RNA weights within each sex to use in differential expression analyses (DEA). Thus, we conducted a DEA with the function “DESeq” [100] using the interaction between sex and scaled RNA weight as the explanatory variables, modeled as “~ sex + sex: scaled_RNA”. Briefly, the “DESeq” function wraps the steps of DEA (estimation of size factors, estimation of dispersion, negative binomial GLM fitting, Wald test). To identify (1) genes with sex-biased expression, (2) genes with expression correlated to size in worms of each sex, and (3) genes with relationships between transcript abundance and size that differ in direction between males and females, the fitted model was input into the “results” function of the DESeq2 package [100] with alpha = 0.05 as the FDR cutoff for the independent filtering. This function extracts the results of the Wald tests for the specified coefficients or contrasts and computes the adjusted p-values using the Benjamini-Hochberg method as in [103, 104] to correct for multiple testing. We used the package EnhancedVolcano [105] and log_2_(fold changes) transformed with the adaptive shrinkage estimator from the package “ashr” [106] to visualize the results of DEA. Venn diagrams were plotted with the R package “venn” [107].

### Gene ontology enrichment analysis

We conducted hypergeometric GO enrichment analysis on the metazoan core orthologs missing from the transcriptome according to the BUSCO analysis, by adapting scripts published by [108], which involved the R packages “clusterProfiler” [109] and “enrichplot” [110]. This allowed us to assess whether there were particular patterns of ortholog loss.

For Gene Ontology (GO) enrichment analyses on DEA results, we combined the GO terms as retrieved by alternative annotation strategies, with the following order of priority: preference was given to InterProScan annotations; if there was none, we queried the eggNOG database for an annotation. If there was neither an InterProScan nor an eggNOG annotation, we searched the SwissProt and TrEMBL databases for potential homologs to *N. agilis* transcripts. We then ran the GO_MWU script by [111], which performs the Mann-Whitney U (MWU) test on log_2_(fold change) ranks. We summarized the results with the list of “best GOs” that this script produces by cutting the clustering tree at h = 9. The MWU approach based on log_2_(fold change) allows to avoid reliance on an arbitrary p-value threshold, and accounts for the directionality of the differential expression.

### Expression of genes involved in meiosis and potential evidence for sexual maturity

Hanson et al. 2013 [111] compiled an inventory of 89 genes involved in meiosis in model species. We searched for transcripts in the *N. agilis* assembly that were homologs of these genes. We filtered the hits to retain only those with a bitscore higher than 100. We then evaluated whether these genes were differentially expressed in relation to the worm’s total RNA weight (as a proxy for size and age).

Finally, we aimed to approximate the size at which most worms would be mature by visually assessing whether there was a change of slope in the relationship between size and transcript expression. For this, among all the *N. agilis* transcripts that were significantly associated with total RNA weight, we selected those that were most significantly so and the most highly expressed, using arbitrary thresholds: average count >1000 for females, >2000 for males; p-values < 0.05 for females, < 0.001 and 0.00001 for males, for positive and negative correlations, respectively. We plotted their counts as a function of total RNA weight, and added a smoothed curve using the “loess” method of the geom_smooth function [112] to illustrate the trends. The point of the arbitrary thresholds was to facilitate visualization by showing fewer than ten transcripts per plot (resulting in between nine and four transcripts).

## Results

### Genome and transcriptome assemblies

The assembled scaffold-level genome resulted in 102 contigs with a total length of 46.2 Mbp, with an N50 of 16.4 Mbp (supplementary Tables S1 and S2 for all relevant metrics). Three major contigs accounted for 94.26% of the total sequence, and the k-mer completeness has been evaluated to 96.35% when using the high-accuracy Illumina reads as a reference for comparison (supplementary Fig. S2). When evaluating the genome assembly using the MetaEuk gene finder within BUSCO, the genome contained 47.1% of the metazoan orthologs and 58.4% of the eukaryotic orthologs from the BUSCO database, while missing 44.7% and 27.9% respectively (complete BUSCO scores: C: 47.1% [S: 45.3%, D: 1.8%], F: 8.2%, M: 44.7%, n: 954 and C: 58.4% [S: 55.7%, D: 2.7%], F: 13.7%, M: 27.9%, n: 255, respectively). BRAKER found 14,442 transcripts (13,655 genes) in the genome using mRNA-Seq evidence. Coding sequences represented 31.6% of the entire genome length.

Separate mRNA-Seq of 74 specimens of *N. agilis* (Eoacanthocephala) resulted in an average of 15.5 million reads per sample (range: 10.5–23.3 million). There was a total of almost 2.1 billion paired reads left after quality filtering. The assembled transcriptome had 123,039 transcript contigs, which Trinity grouped into 41,888 genes. It should be noted that Trinity “genes” can also be variants of single genes, so that the actual gene number will be smaller. Transrate recognized 42,527 transcripts with ORFs (Table 2). Matching the latter number with the BRAKER estimate on gene number, there were roughly three transcript variants per coding gene in *N. agilis* on average.

**Table 2:** Transcriptome metrics as derived with transrate

Metric	Value (lengths in bases)
Number of transcripts	123,039
Minimum transcript length	181
Maximum transcript length	24,466
Total number of bases	198,678,817
Mean sequence length	1,614.72
Number of transcripts under 200	29
Number of transcripts over 1 kb	57,429
Number of transcripts over 10 kb	812
Number of transcripts with ORF	42,527
Mean ORF percent	32.87
GC content	0.39

**Fig. 1:**
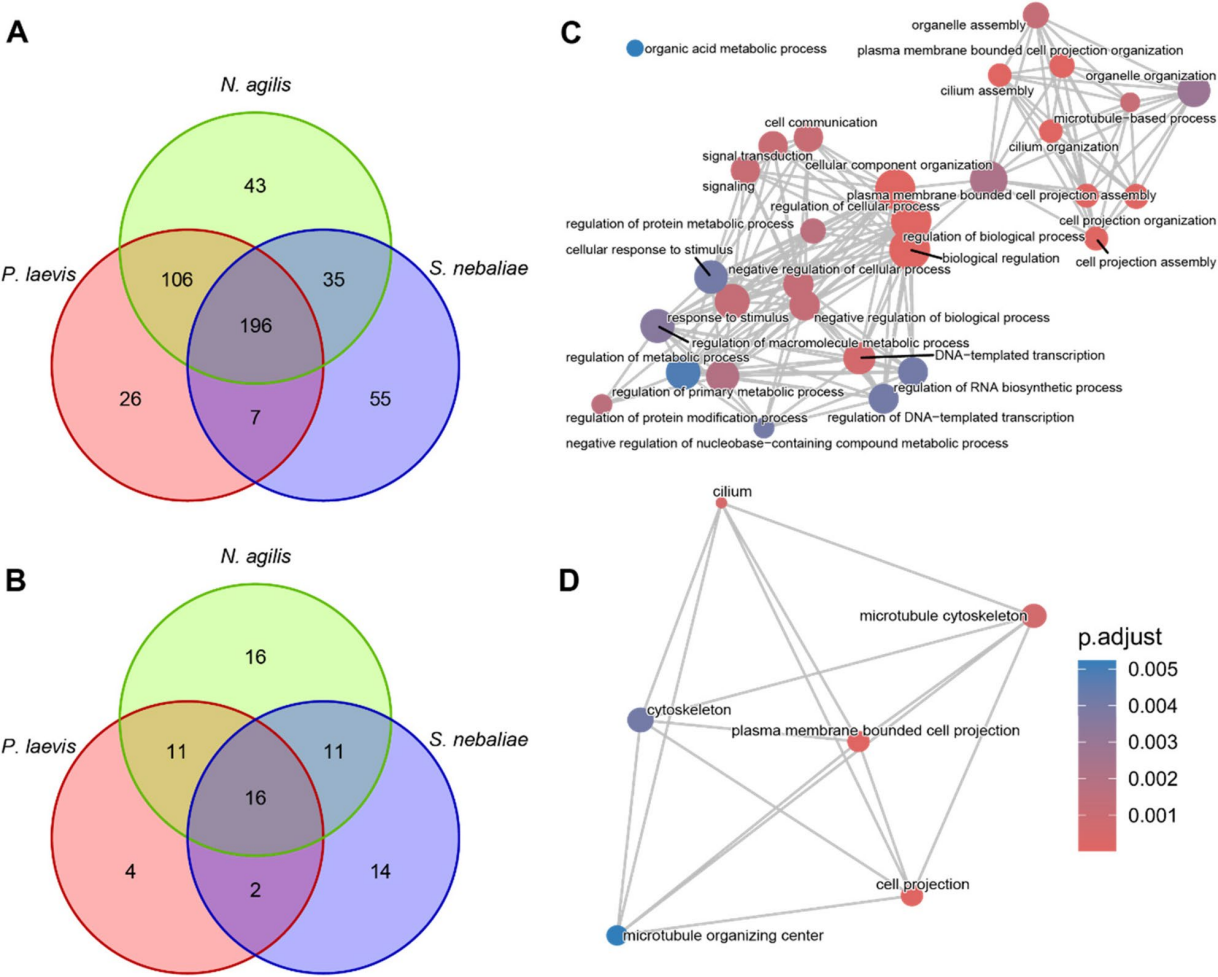
BUSCO missing from the transcriptome of *N. agilis*. Left: number of missing (**A**) metazoan and (**B**) eukaryotic core orthologs identified through BUSCO in the transcriptomes of *N. agilis*, *P. laevis*, and *S. nebaliae*. Right: enrichment map of GO terms over-represented amongst metazoan core orthologs missing from the *N. agilis* transcriptome for (**C**) Biological Process and (**D**) Cellular Component. The colors represent p-values adjusted after Benjamini-Hochberg correction [103, 104] of the one-sided Fisher’s exact test p-values. The size of the nodes is proportional to the number of missing orthologs they contain

There were 54.9% of the metazoan core orthologs present in the transcriptome, whereas 40% of these were lacking (C: 54.9% [S: 7.2%, D: 47.7%], F: 5.1%, M: 40.0%, n: 954). In addition, 71% of the eukaryotic core orthologs were represented in the *N. agilis* transcriptome, while 21.5% were missing and 7.5% were fragmented (BUSCO score C: 71.0% [S: 10.2%, D: 60.8%], F: 7.5%, M: 21.5%, n: 255). Among the 381 missing metazoan BUSCO orthologs and 55 missing eukaryotic orthologs (Tables S3 and S4), 365 (95%) and 48 (87%), respectively, were not detected in any of the 74 individual assemblies screened. In addition, we found that 79% of missing eukaryotic core orthologs were also absent from the transcriptome of *P. laevis*, and 61% were also missing from the transcriptome of *S. nebaliae*. The corresponding proportions of shared missing metazoan core orthologs were 50%, both for *P. laevis* and *S. nebaliae* (Fig. 1A, B).

Results of the hypergeometric test revealed several biological processes that were over-represented in missing metazoan core orthologs, which can be grouped in two main modules (Fig. 1C, D). The first one groups terms associated with “plasma-membrane bounded cell projection”, in particular cilium assembly and organization. The second group clusters several terms descending from “regulation of cellular process”, “regulation of biological process”, and “cellular component organization”. More specifically, several terms in this second group were linked to signaling, response to stimulus, and cell communication, or the regulation of macromolecule metabolic process (Fig. 1C, D).

### The translated proteome

We kept the 104,397 transcript contigs that Transrate had classified as “good”. This was the case in 85% of the initial 123,039 contigs. None of the eukaryotic and only a single metazoan core ortholog was lost upon this filtering step. Of the kept transcripts, 54,003 bore ORFs and hence could be translated. InterProScan detected domain signatures in 41,049 predicted proteins (76%), and the eggNOG mapper annotated 33,667 predicted proteins (62%). DIAMOND recognized homologs with e-values < 0.001 for 29,755 (55%) and 35,423 (66%) predicted proteins in the SwissProt and TrEMBL databases, respectively. Altogether, we were able to assign functional annotations to 43,440 transcripts, or 12,247 genes *sensu* Trinity. Among these genes, 9409 were annotated with GO terms. Detailed annotations for single transcripts are reported in the supplementary material (supplementary Tables S5 to S8).

**Fig. 2:**
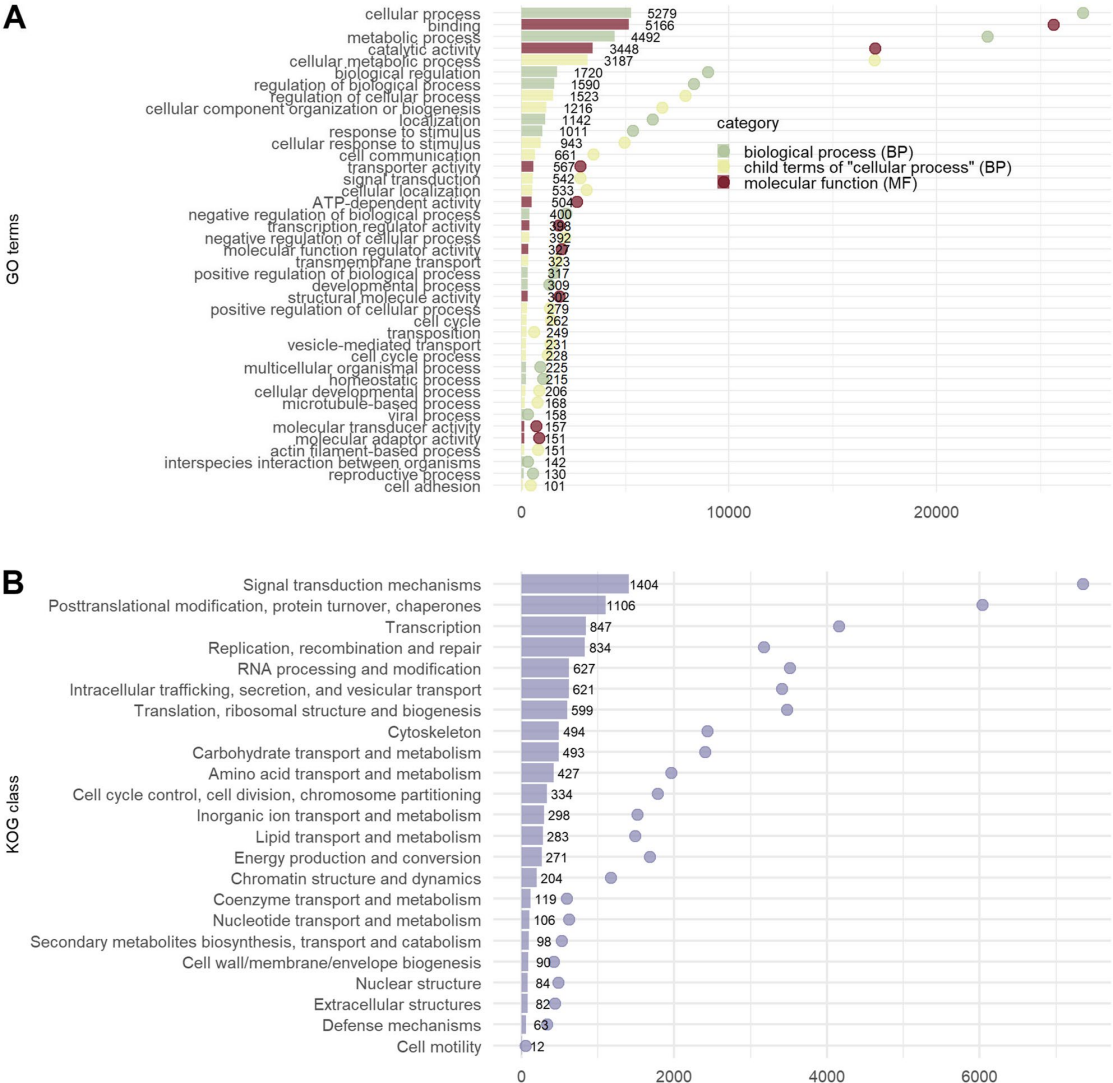
Summary of the functional involvements of the *N. agilis *transcriptome. The bars indicate the number of assembled transcripts annotated with (**A**) child terms of “biological process” (in olive green), child terms of “cellular process” (the most frequent “biological process”, in yellow), and child terms of “molecular function” (in red-brown) or (**B**) the KOG classes in the bottom panel. The points represent the sum of the mean variance-stabilized abundances of all the transcripts annotated with the GO terms or KOG classes. The legends list the terms in decreasing order of their representation in the transcripts. Only the GO terms represented by more than 100 transcripts are shown

The majority of Trinity “genes” were annotated with Biological Process GO terms descending from “cellular process” and “metabolic process” (Fig. 2A). The most prevalent direct child terms of “cellular process” were “cellular metabolic process”, followed by “regulation of cellular process” and “cellular component organization or biogenesis”. Additional prominent GOs were “biological regulation” and “regulation of biological process”. The most prevalent Molecular Function GOs in the proteome were “binding” and “catalytic activity”. The KOG classes with the most genes were “Signal transduction mechanisms” and “Posttranslational modification, protein turnover, chaperones”, followed by “Transcription” and “Replication, recombination and repair” (Fig. 2B).

### Sex determination in transcriptomic datasets of *N. agilis*

In preliminary PCA and heatmap based on variance-stabilized transcript counts, most specimens clustered together with other specimens that were assigned the same sex. Yet, seven worms grouped with clusters mostly composed of datapoints from the opposite sex (supplementary Figs. S4 and S5). Sample-to-sample clustering based on similarity in expression of genes that were found sex-biased in another acanthocephalan species (*P. laevis* [102]), provided additional supportive evidence that five individuals were probably misclassified as females and two were misclassified as males (see supplementary methods and Fig. S6). These samples were eliminated, and we focused on the remaining 66 *N. agilis* specimens (30 females, 36 males).

**Fig. 3:**
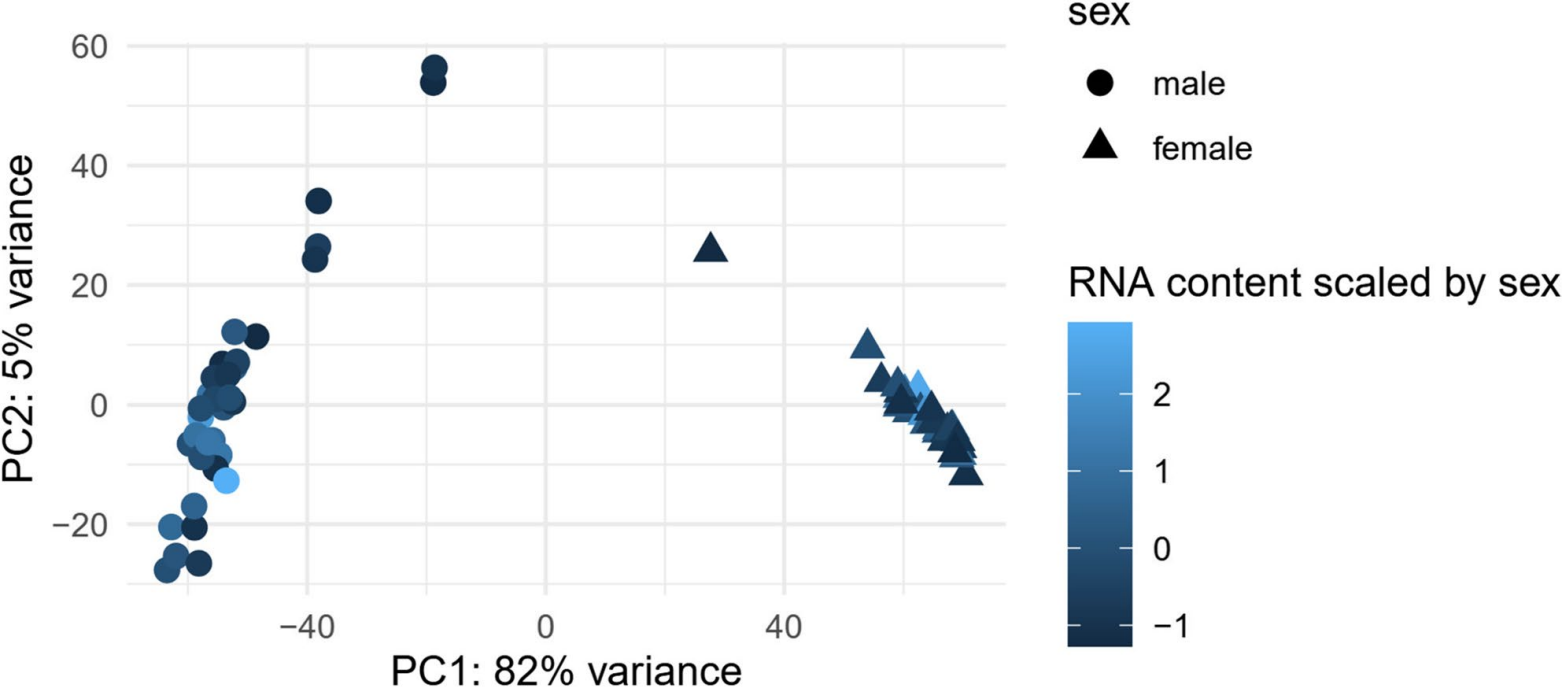
Principal Component Analysis (PCA) of 66 *N. agilis* transcriptome profiles based on variance-stabilized abundances. Triangles and circles represent females and males, respectively. Colors indicate total RNA weight, scaled within sexes to account for sex size dimorphism

Overall, higher amounts of RNA were extracted from females than from males. We isolated between 417 and 7250 ng total RNA (mean ± sd = 2583 ± 1733 ng) from female worms kept after filtering, and between 46 and 3535 ng (mean ± sd = 1104 ± 832 ng) from retained males. This corresponds to a ratio of female to male weight of 2.34. The PCA based on the entire expression profiles of these 66 individuals further showed separate clusters for males and females along axis PC1, which represented 82% of the variance (Fig. 3). An additional 5% of variance was covered by PC2 and 2% by PC3. Small worms’ loadings on PC2 were more dispersed than those of bigger worms, which was confirmed by plotting the PC2 loadings against the scaled RNA content and testing for heteroscedasticity in the regression between the two (Breusch-Pagan test: BP = 5.01, p = 0.025; supplementary Fig. S8).

### Differential expression analysis

#### Sex-biased gene expression

**Fig. 4:**
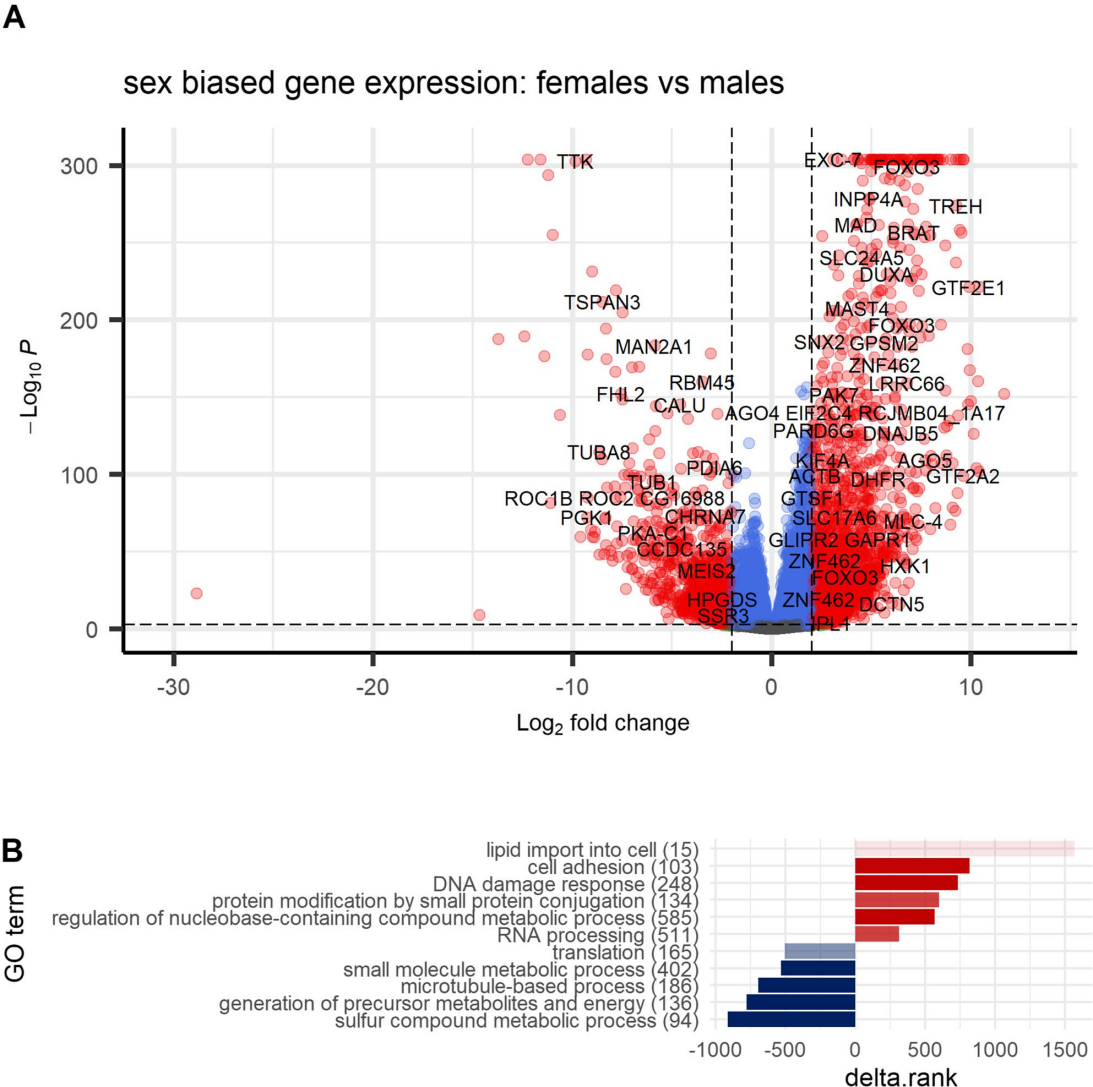
Sex-biased gene expression in *N. agilis*. (**A**) Volcano plot showing log_10_(p-values) in relation to shrunken log_2_(fold changes) as results from differential expression analysis between male and female *N. agilis* specimens. Transcripts with positive log_2_(fold changes) are upregulated in females compared to males and vice versa. Colored points represent transcripts differentially abundant with an adjusted p-value < 0.001; red points represent transcripts differentially abundant with a log_2_(fold changes) >2 (blue: < 2; cutoffs chosen for the sake of readability). Transcripts are labeled with gene names from eggNOG annotation, or from the best hit from blastp (DIAMOND) annotation against the UniProt database when no eggNOG annotation existed. (**B**) Rank-based Gene Ontology (GO) enrichment analysis for Biological Processes in females compared to males. The ranking is based on log_2_(fold change) and significance, assessed with the Mann-Whitney U test, corrected for multiple testing using the Benjamini-Hochberg method [103, 104]. Colors represent the rank sign: red terms have a positive mean rank, meaning the genes annotated with them are, on average, upregulated in females. Blue represents male-biased terms. The opacity is proportional to the adjusted p-value (darker colors represent lower p-values). For summarization, we only show “best GOs” (“GO terms that best represent independent groups of significant GO terms”, as defined by [111] in their GO_MWU script), computed by cutting the tree at height 0.9, using an adjusted p-value cutoff of 0.01 on representative GO terms. The full results are reported in the supplementary Table S9 and Figs. S9 and S10

Applying an adjusted p-value threshold of < 0.05, the number of transcripts with higher abundances in males than females (*N* = 6199, 15%) was similar to the number of female-biased transcripts (6114, 15%). These will thereafter be referred to as male-biased, female-biased or, collectively, sex-biased genes. The extent of female expression bias was overall stronger (median log_2_(fold change) = 1.5) than it was in male-biased genes (median log_2_(fold change) = 1; Fig. 4A, supplementary Table S9). About 50% (6138) of sex-biased genes had received functional annotation.

Biological Process GO terms significantly enriched with female-biased genes can be, for the most part, categorized into “cell adhesion”, “DNA damage response”, “protein modification by small protein conjugation”, “regulation of nucleobase−containing compound metabolic process”, “RNA processing”, and “lipid import into cell” (Fig. 4B, supplementary Figs. S9 and S10). Male-biased transcripts enriched GO terms that can be grouped into “sulfur compound metabolic process”, “generation of precursor metabolites and energy”, “microtubule-based process”, “small molecule metabolic process”, and “translation” (Fig. 4B, supplementary Figs. S9 and S10).

#### Size-associated gene expression

**Fig. 5:**
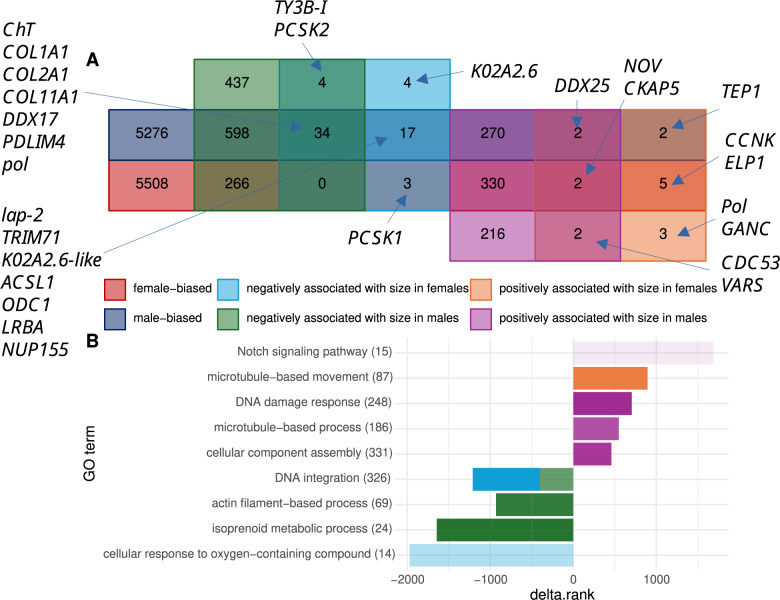
Gene expression in relation to worm size. (**A**) Venn diagram showing the number of genes differentially expressed (p < 0.05); red: female-biased; dark blue: male-biased; orange: positively associated with size in females; purple: positively associated with size in males; light blue: negatively associated with size in females; green: negatively associated with size in males. The proxy for size is total RNA weight. Genes – only for groups with few genes – are indicated at the beginning of the blue arrows; complete results are reported in supplementary Table S9. (**B**) GO enrichment as in Fig. 4B. Positive delta.ranks indicate that the GO term is enriched with genes positively associated with size, and inversely. The opacity is proportional to the adjusted p-value (darker colors represent lower p-values). The numbers next to the terms report the number of genes annotated with it. The full results are in supplementary Fig. S11

DEA also revealed 822 transcripts that displayed a significant (adj. p < 0.05) linear rise in abundance with increasing male worm size, while a negative association with male size was observed in 1339 transcripts (supplementary Table S9). Corresponding numbers were lower in females, with 16 positive and 62 negative correlations between abundance and size (supplementary Table S9). Furthermore, 1630 (75%) of the male and 54 (69%) of the female size-associated genes received an annotation. Size-correlated transcripts were partly shared between males and females: 6 and 38 transcripts were positively, respectively negatively, correlated with size in worms of both sexes (Fig. 5A).

Among the six transcripts positively correlated with size in both groups, five received a protein annotation: ATP-dependent RNA helicase encoded by *DDX25*, cell division control protein 53 (*CDC53*), valine-tRNA ligase (*VARS*), protein NO VEIN (*NOV*), and cytoskeleton-associated protein 5 (*CKAP5*). To note, transcripts homologous to *NOV* and *CKAP5* were also found to be female-biased, and *DDX25* was male-biased (Fig. 5A).

In females, the most strongly enriched GO term for genes positively correlated with size is “microtubule-based movement”. In males, transcripts with positive abundance-size association enriched “Notch signaling pathway”, “DNA damage response”, “microtubule-based process”, and “cellular component assembly” (Fig. 5B and supplementary Fig. S11).

Nine out of the 33 transcripts negatively associated with size in both sexes were annotated. Expression of the homologs of transposon Ty3-I Gag-Pol polyprotein (*TY3B-I* gene) and neuroendocrine convertase 2 (*PCSK2*) was not sex-biased. The seven others were male-biased regarding their expression. These were probable ATP-dependent RNA helicase (*DDX17*), collagen alpha-1(I), alpha-1(II) and alpha-1(XI) chains (*COL1A1*, *COL2A1* and *COL11A1*), high-affinity choline transporter (*ChT*), Gag-Pol polyprotein (*pol*), and PDZ and LIM domain protein 4 (*PDLIM4*) (Fig. 5A). In females, “DNA integration” and, to a lesser extent, “cellular response to oxygen-containing compound” summarized GO terms enriched with transcripts negatively associated with size. Transcripts negatively associated with size enriched “isoprenoid metabolic process”, “actin filament-based process” and “DNA integration” in males (Fig. 5B and supplementary Fig. S11). By contrasting the log_2_(fold changes) of males and females, we found that only four transcripts had significantly different abundance-size relationships. Three of them were annotated. A homolog of LINE-1 retrotransposable element ORF2 protein (*Pol*) and a homolog of Cyclin-K (*CCNK*) had a non-significant negative correlation with size in males and a significant positive association with size in females, and Importin-4 (*IPO4*) was strongly positively correlated with size in males and had no association with size in females.

#### Expression of genes involved in meiosis and potential evidence of sexual maturity

**Fig. 6:**
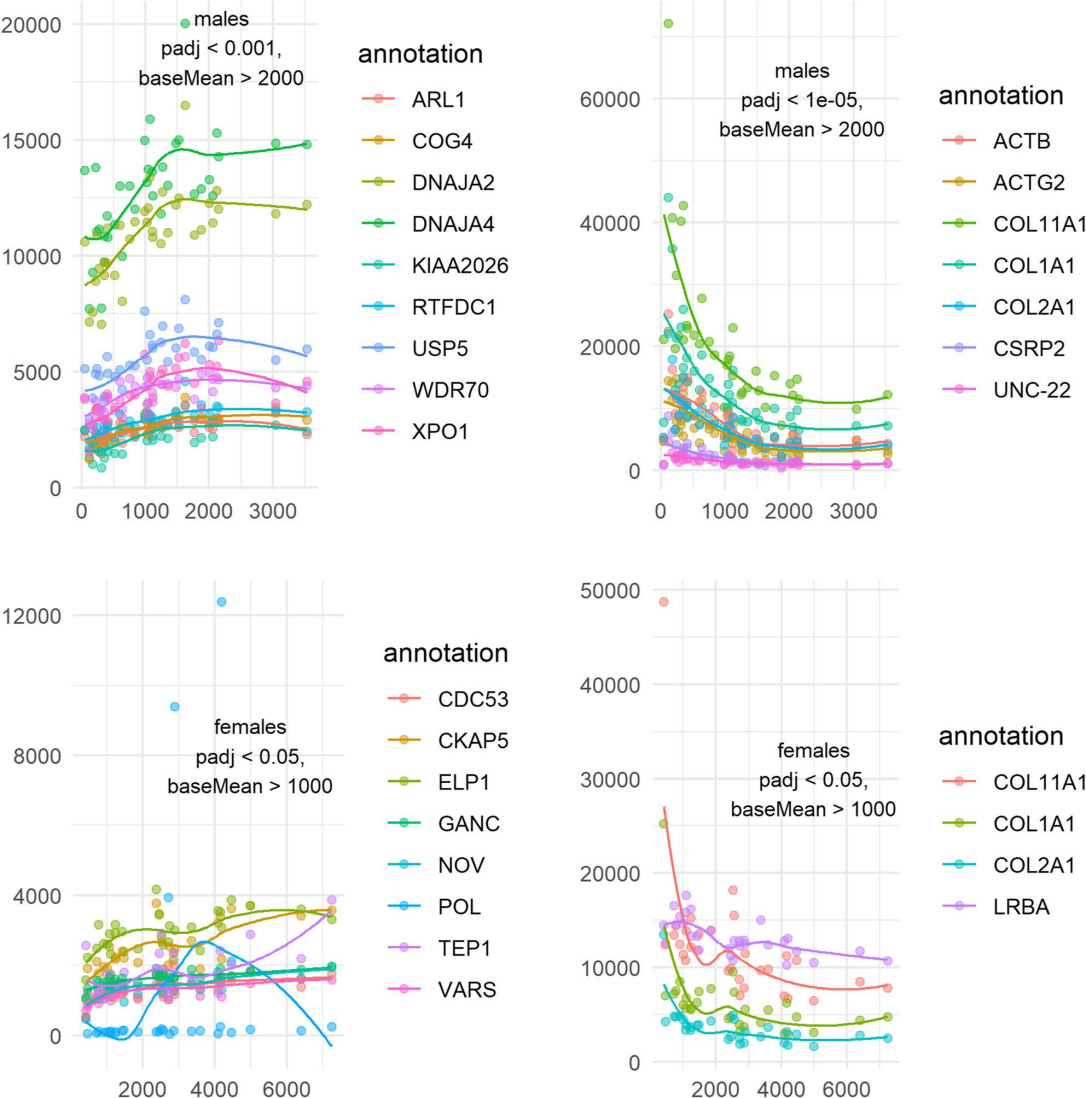
Expression of genes correlated to size. Variance-stabilized counts are shown in relation to whole body RNA weight in males (top plots) and females (bottom plots) in nanograms. The plots on the left show counts of genes positively associated with RNA weight, and the plots on the right show counts of genes negatively associated with RNA weight. Thresholds written on the plots were selected for each category to show fewer than ten transcripts per plot. The curves were plotted using geom_smooth to visualize trends and do not represent the DESeq2 model coefficients. Color codes refer to proteins as encoded by respective transcripts

By plotting the most differentially expressed transcripts in relation to size (Fig. 6) for males and females, a trend appeared for male worms: transcripts increase or decrease in abundance in relation to whole-body RNA weight, until reaching a plateau. The slopes flatten in worms of more than 1000–1500 ng. A similar trend existed in transcripts negatively associated with size in females: the negative association between RNA weight and gene counts is strong in small worms, but, for some genes, flattens in worms of more than 1500 ng. This represents a smaller proportion of worms in females.

**Fig. 7:**
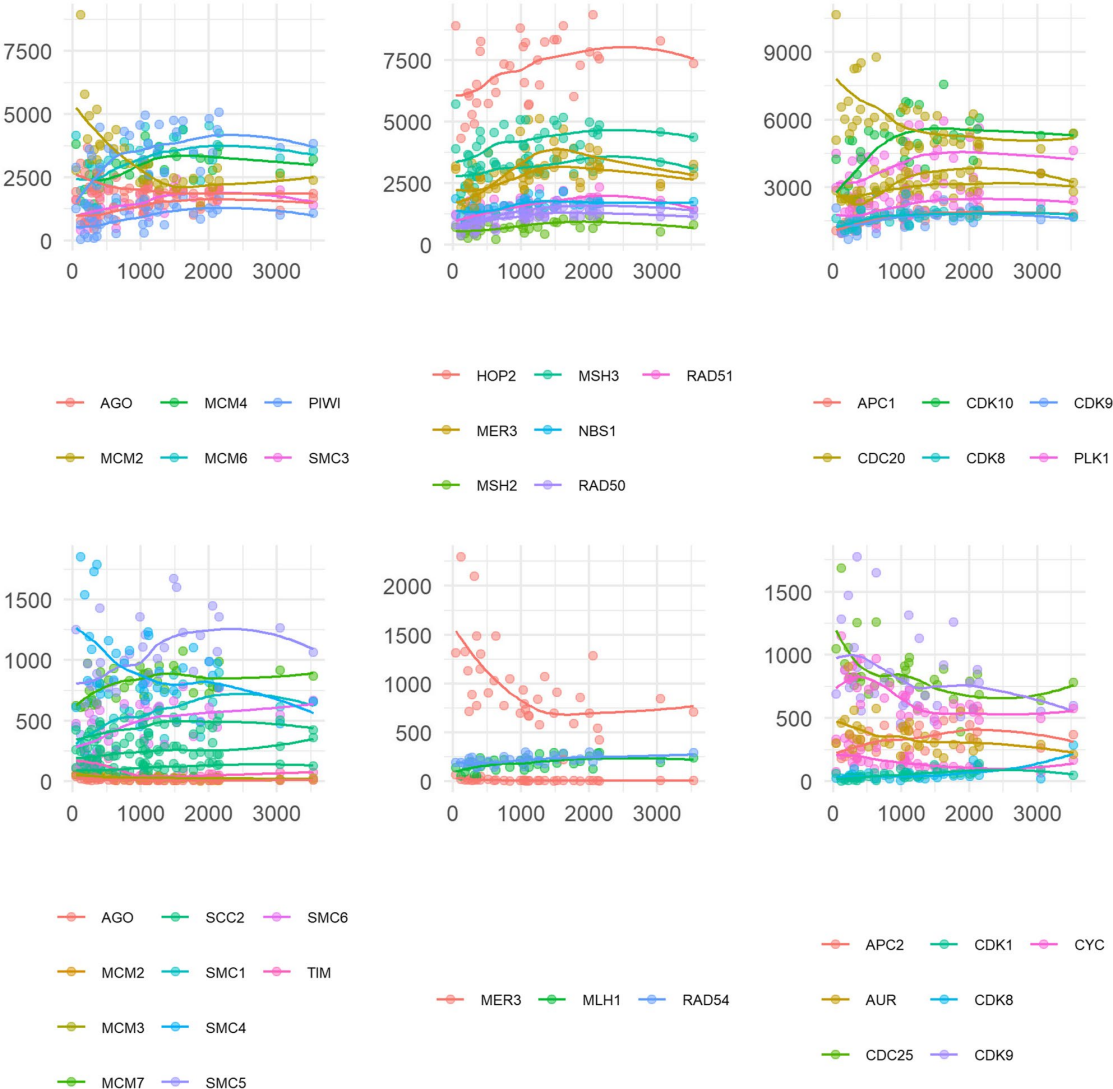
Expression of meiosis-related genes. Variance-stabilized counts are shown in relation to male whole-body RNA weight in nanograms. The genes presented here were gathered in an inventory by Hanson et al. [113] because they play a role in meiosis in model organisms. They were further categorized as involved in meiosis entry, DNA replication, and chromosome structure (left), meiotic recombination (middle), and meiosis progression (right). Only transcripts that were significantly (p < 0.05) correlated with total RNA weight are shown. The curves were plotted using geom_smooth to visualize trends and do not represent the DESeq2 model coefficients. Genes with average variance-stabilized counts > 1000 are shown on the top, and those with < 1000 are shown in the bottom, for the sake of readability. Color codes refer to proteins as encoded by respective transcripts

In the *N. agilis* transcriptome annotation, we detected 64 of the meiosis-related genes from the inventory curated by Hanson et al. [113]. None of them was significantly associated with size in females, but 33 were associated with size in males, among which 26 showed a positive correlation. Again, expression of several meiosis-associated genes decreased or increased with size in the smallest half of the worms, before “flattening” or changing direction (Fig. 7). For several genes, this trend seemed to be driven by the two largest worms, which yielded more than 3000 ng of RNA (see supplementary Fig. S12, where these two outliers were removed), and would otherwise be monotonic. For instance, DNA replication licensing factor (*MCM2 *gene) became less abundant as the worms were larger and then stabilized. On the other hand, most meiosis-related genes, Cyclin-dependent kinase 10 (*CDK10*) most strikingly, showed an increase in abundance followed by a plateau in worms with more than 1000 ng of RNA, even after removal of the two largest worms.

Finally, cell division cycle protein 20 (*CDC20 *gene) and ATP-dependent DNA helicase homolog (*MER3*) showed diverse, opposing abundance-size associations. Indeed, several transcripts were annotated as their homologs. These different expression patterns might indicate isoforms with different effects or regulation patterns, or simply different genes that are homologs but distinct.

### Results in a nutshell

In *N. agilis*, the nuclear genome spans about 46.2 Mb, in which 13,655 coding genes might reside. The coding portion occupies about 32% of the entire genome. Genome compactness is additionally reflected in the absence of significant proportions of eukaryotic (22%) and metazoan (40%) core orthologs. Except for the absence of ciliary involvements, functions highlighted by GO terms over-represented among missing metazoan core orthologs were, for the most part, still represented in the transcriptome. Functional annotations underscored the predominance of transcriptional and cell division activity in females. In turn, energy metabolism and microtubule-based processes are prevalent tasks throughout male life (Fig. 4B).

## Discussion

### The nuclear genome and transcriptome of *N. agilis*

The nuclear genome assembly of *N. agilis* shares several features with phylogenetically close relatives within the clade comprising traditional rotifers and acanthocephalans [1–6]. First, nuclear genome size appears to be very similar in *N. agilis* (46.2 Mb) and a member of the likely acanthocephalan sister-group, *S. nebaliae* (44–46 Mb), although five times smaller than reconstructed for the palaeacanthocephalan *P. laevis* [5, 78, 114,]. Furthermore, the number of coding genes (13,655) determined via RNA read-mapping in the *N. agilis* genome draft is in a likewise range as in *P. laevis* (12,073). Also, the number of *N. agilis* transcripts with ORFs (42,527) is close to the number of transcript contigs assembled for *P. laevis* (42,888; [77]). The compactness of the *N. agilis* genome (coding portion: 31.6%) is somewhat smaller than in *S. nebaliae* (45%), although seemingly higher than in *P. laevis* [114]. We take these consistencies as a confirmation of the representative character of present genome and transcriptome assemblies for *N. agilis*. In line with this, the lack of a significant portion of usually conserved eukaryotic and metazoan core genes seems to be common in acanthocephalans and their closer phylogenetic relatives [114, 115]. Most probably, this reflects repeated gene loss upon the delegation of functions to the host, in acanthocephalan evolution [77].

Gene loss, function loss, genome compaction, and unique adaptations, although not systematically surveyed, have been repeatedly observed in other groups of parasites [108, 116–118]. Our investigation of missing metazoan core orthologs in the transcriptome of *N. agilis* showed an enrichment of the general GO terms “regulation of cellular process”, “biological regulation”, and “regulation of biological processes” (Fig. 1), as already highlighted for *N. agilis* and *S. nebaliae* genomes [115]. More specific GO terms in the transcriptome of *N. agilis* are compatible with gene loss in the functional domain of sensing. In particular, it seems to lack metazoan orthologs associated with signaling, response to stimulus, and cell communication. In addition, missing orthologs enriched numerous terms associated with “plasma membrane bounded cell projection”, and more specifically, cilia, which functions usually cover movement and sensory perception. This absence is, however, unsurprising since acanthocephalans are known to generally lack cilia, except for the excretory tubules of certain species to which *N. agilis* does not belong [119]. Notably, the most strongly enriched categories of GO terms for missing metazoan orthologs were very similar to those missing in the genomes of two nematomorph parasites [108]. Indeed, the missing BUSCOs of the horsehair worms also enriched the terms “regulation of biological process”, “regulation of cellular process”, and “biological regulation”. In addition, the same GO terms associated with cilia were enriched with missing metazoan core orthologs, which was also straightforwardly explained by the apparent absence of cilia in Nematomorpha [108]. Overall, functions associated with missing orthologs in *N. agilis* might reflect the simplification of sensory pathways in a parasite that might not need to perceive and react to a wide variety of cues, compared to the free-living metazoans from which the BUSCO database was composed. Furthermore, the sensory organs of acanthocephalans are relatively simple and seem to be mostly restricted to the lateral sensory pores and/or the apical sensory pores, depending on the species [120–123]. These sensory organs are dendritic endings embedded in protrusions of a support cell (“Stützzelle” [121]), and are located at the neck (lateral sensory organs) or on the apex (apical sensory organs) of the proboscis. The males of some species also have sensory cells on their bursa [124]. *N. agilis* harbors lateral sensory organs [125] as well as sensory papillae on the bursa of male worms [126]. However, microtubule arrangements suggestive of cilia have not been reported for any of these sensory differentiations.

Energy metabolism is central for the development of any organism, but might be even more so for parasites tailored to high fecundity. In acanthocephalans, the resulting high energy demand is met by simultaneous respiration and fermentation pathways delivering energy and reduction equivalents [127–130]. Acanthocephalans are even said to ferment in the presence of oxygen [129–131]. This metabolic duality reflects that the trunk is exposed to the oxygen-deprived intestinal lumen [132] while the hooked attachment organ, the proboscis, can damage the intestinal wall of the host, thus getting into contact with oxygenized blood [24, 30, 31]. During larval development, acanthocephalans reside in the hemocoel of arthropods and are thus immersed in the hemolymph, which is oxygenated under normal conditions [133]. The ability to produce ATP under normoxia and hypoxia will be crucial for these parasites. Advantageous will be that Acanthocephala can store excess energy in the form of glycogen granules deposited in the cytoplasmic parts of the musculature [25], which can be utilized in the event of energy shortage under varying oxygen regimes [134, 135]. Broader phylogenetic comparison shows that the capability of fermentation and respiration is not restricted to acanthocephalans. Rather, a corresponding condition is seen in tapeworms (Platyhelminthes: Cestoda) [136, 137], which additionally share the lack of a digestive tract and nutrient uptake via the body surface [138]. Cestodes and acanthocephalans thus have in common strong reliance on host metabolites [139–142], which is further reflected by the loss of orthologs in metabolic pathways (Fig. 1 [115, 137, 143–145]),.

On a final note, despite low BUSCO scores being most likely caused by gene loss, it cannot be fully excluded that technical issues (such as insufficient sequencing depth or assembler limitations) partially caused low completeness of the assemblies. Nevertheless, the fact that the vast majority of core orthologs missing from the main transcriptome assembly were also missing from all the transcriptomes assembled from individual libraries (= specimens) tends to underline a reduced gene repertoire in *N. agilis* rather than a high incompleteness of the assembly.

### Male and female peculiarities of transcriptome profiles in *N. agilis*

Distinct male and female transcriptome profiles, as demonstrated here for an eoacanthocephalan (*N. agilis*), were previously reported for the palaeacanthocephalan *P. laevis* [39, 102]. This is in agreement with marked sex-specific differences in the internal organization of the trunk, which usually contributes the most to body size in acanthocephalans. In males, the trunk usually bears two large testes arranged in tandem [146]. Further differentiations include one larger or several smaller glands producing a proteinaceous secretion by which the male seals the female genital tract after copulation [147]. There are also efferent ducts and and the aforementioned eversible bursa for grasping the female hind end, and a penile structure for internal fertilization [45, 148, 149]. In the female, there is a peculiar egg-sorting apparatus (uterine bell) through which only mature eggs pass from the body cavity into the uterus and vagina, from where they are discharged into the host’s digestive tract [50, 150–152]. While the female genital tract occupies comparably little space, the largest part of the trunk lumen is fraught with numerous free-floating ovaries and, upon insemination, embryonated eggs [149, 153, 154]. This pronounced morphological divergence is consistent with the distinctiveness of male and female expression profiles and the high proportion of sex-biased genes (30% with p < 0.05) in the present study.

### Anchoring RNA amounts in body size

Sexual dimorphism was additionally reflected in overall larger RNA amounts extracted from female than male worms. In fact, acanthocephalan females are known for growing larger than males (e.g [7, 8]). In *N. agilis*, there is consistent evidence for sexual size dimorphism. In [51], females were found to be only slightly larger (mean length ~ 8 mm) than males (~ 7 mm), whereas measurements from other investigations indicated much stronger size differences. Saleh et al. [126] recorded maximum trunk lengths of 18.23 mm in females and 8.12 mm in males, which corresponds to a female-male size ratio of 2.245. Likewise, Tepe et al. [155] reported mean body lengths as measured from the tip of the proboscis to the rear end of the trunk with 5.643 mm (range: 4.121–7.511 mm) in males and 12.495 mm (4.852–26.126 mm) in females of *N. agilis*. According to the latter, female worms would get 2.214 times as long as their male counterparts on average. Both size ratios derived from previous studies are very close to the ratio between the means of total RNA amounts which we had extracted from female and male worms (= 2.34). We take this as an affirmation that individual RNA amount gives a reasonable approximation of animal size and developmental maturity in *N. agilis*, even though the relationship between body size and total RNA weight might not be linear.

### Gene functions in acanthocephalan adults

The GOs enriched with sex-biased transcripts underscored a strong focus on reproduction in adult *N. agilis* specimens. Thus, a noticeable number of GOs linked with male-biased transcripts were associated with microtubules. This fits the above mention that male acanthocephalans have disproportionately large testes. These should produce large amounts of sperm each carrying a flagellum, the motility of which is enabled by microtubule-based movement [156]. As mentioned above, *N. agilis* and most other acanthocephalan species seem to lack motile cilia other than sperm flagella. Furthermore, the testes occupy a significant proportion of the trunk body cavity soon after infection of the definitive host and continue growing in the process of maturation [45, 46]. It is therefore not surprising that microtubule-related terms were enriched among transcripts with male-biased expression. In addition, acanthocephalan testes will be organs of intense cell division activity. In line with this, some of the transcripts with highly male-biased abundances in *N. agilis* can be directly linked with the spindle apparatus, such as the dual specificity protein kinase (gene: *TTK*, or *MPS1* for monopolar spindle 1) [157]. Others play established or suspected roles in sperm morphogenesis and motility, such as dynein regulatory complex subunit 7 (*CCDC135*) in mice [158] or phosphoglycerate kinase 1 (*PGK1*) in humans [159]. Additional functional enrichments among transcripts with male-biased abundances referred to various metabolic processes, particularly energy metabolism, which is important for any organism. Sperm production might sustain an even higher energetic cost in species such as *N. agilis*, which provision sperm with so much glycogen that mature sperm still carry reserves [160]. Glycogen from mature sperm disappears after penetration into the female body cavity [160].

Overrepresentation of GOs linked with metabolic processes, energy supply, and conversion in transcripts with male abundance bias could also reflect competition between males. Competition between male acanthocephalans for reproductive success is evident from the large size of their testes (relative to body size), which should enable the production of large quantities of sperm [161]. The fact that functional sperm already occur in the cystacanth stage underlines strong competition among male individuals, whereby early onset of gonad differentiation could imply a selective advantage [43, 162]. Male acanthocephalans even develop larger testes when they encounter more competitors [46]. Also, the sealing of the female genital tract post copulation can be interpreted as a male counterstrategy aimed at preventing subsequent insemination by competitors [163, 164]. However, the production of cement and high quantities of sperm is costly, and male reproductive success will depend on occupying favorable positions in the gut, providing sufficient energy and access to female mating partners [165, 166]. If better sites are already occupied, males would be forced to settle for energetically less favorable positions along the host gut. In fact, larger size likely confers a competitive advantage, at least in the context of ensuring proximity to females as outlined in [167]. Since *N. agilis* females grow larger than males (see above), they may be more successful in occupying the best sites. Consistent with this scenario, more female than male *Neoechinorhynchus* specimens were noticed in their fish hosts, and larger worms predominated in the beneficial posterior section of the gut [168].

The functions overrepresented among female-biased transcripts – such as DNA damage response, regulation of transcription, RNA processing, modification of proteins, and cell adhesion – also align well with the huge reproductive investment in females, including the formation of thousands of ovarian balls and numerous eggs produced upon insemination, each containing a developing or fully differentiated acanthor [150, 154, 155]. In larger species, females may release up to 82,000 eggs per day over a period of ten months [48]. The females of the smaller species, such as *N. agilis*, will produce fewer eggs per lifetime, but the number will remain considerable. It is worth noting that DNA damage response is also crucial for early embryos, as harm to genome integrity will have dramatic consequences for organ formation and development in general [169].

The low proportion of transcripts showing significant correlation between abundance and total RNA weight in female *N. agilis* (0.19%, versus 5.40% in males) might indicate that sexual maturation in female *N. agilis* completes rapidly after arrival in a fish’s intestine, and the size of females attached in the final host might not be a strong indicator of their sexual maturity. This is congruent with the observation that none of the 64 meiosis-associated genes from the inventory by Hanson et al. [114] found in the transcriptome of *N. agilis* were associated with size in females, although the majority were represented by female-biased transcripts: the meiotic activity seems stable in females, whatever their size. Alternatively, total RNA weight might be a poor indicator of size and/or age in female *N. agilis*, or the sampling might not cover the entire age range. Still, genes upregulated in bigger females are congruent with heightened reproduction and embryonic development. Indeed, the enrichment of the GO term “microtubule-based movement” in bigger females could be caused by the flagella of hundreds of sperms present in the trunk of mated females, but also by spindle formation during cell division in developing eggs. In fact, mature sperm carry significant amounts of RNA, as was evidenced in vertebrates [169–172] and invertebrates [173, 174]. Still, cell division might be the explanation that most strongly underpins the enrichment of the “microtubule-based process” in females, given that the microtubule-associated gene showing the most significant positive correlation with size codes for cytoskeleton-associated protein 5 (*CKAP5*), involved in spindle pole organization [175, 176]. Finally, it is worth noting the increased abundance of the transcript coding for alpha-glucosidase (*GANC*), which is likely crucial for the breaking down of glycogen in the sperm and the ovarian balls observed in *N. agilis* after insemination [159].

The present study lacks morphometrics and detailed anatomical information, thus allowing us to draw only tentative conclusions on the relationship between size and maturation level of *N. agilis*. Nevertheless, the variance-stabilized counts of the most differentially abundant transcripts hint at a nonlinear relationship between their expression and whole-body RNA weight (Fig. 6). Indeed, increases or decreases in the expression of these genes seem to slow down from a certain size on, especially in males, which had more size-associated genes. Assuming that whole-body RNA weight is linearly correlated with body size, we could use the change in slope to approximate size at maturity. The genes encoding two members of DnaJ homologs subfamily A (co-chaperones of Hsc70) and a homolog of ubiquitin carboxyl-terminal hydrolase 5 (*USP5*) demonstrated this pattern strongly. Transcript abundances here show a positive correlation with size only in the small males, possibly suggesting a response to stressful conditions, or simply the importance of the maintenance of proteostasis in ageing males. In addition, a sharp negative correlation between the expression of genes coding for components of fibrillar collagen types I and II – structural components of connective tissues – and the size of both males and females strongly suggests the deceleration of body growth. This elbow in the abundance-size association also existed in many meiosis-related genes in males (Fig. 7). Thus, we could approximate that female specimens that yielded more than 2 micrograms of whole-body RNA were mostly mature adults, as well as males that yielded more than 1 to 1.5 micrograms of total RNA. Interestingly, the one gene from the inventory by Hanson et al. [113] that is only known to be active during meiosis, *HOP2*, was strongly expressed in male worms of all sizes, and its transcript abundance was correlated with RNA weight. The expression pattern of this transcript, involved in meiotic recombination, suggests that meiosis carries on during the entire acanthocephalan adult life, assuming our sampling covers a wide range of worm ages.

### Key genes

Five of the six transcripts with consistently positive correlations between abundance and size in males and females received detailed annotations. One of these transcripts might code for the ATP-dependent RNA helicase DDX25. This DEAD-box RNA helicase, also known as gonadotropin-regulated testicular RNA helicase (GRTH), acts as a transporter of specific mRNAs from the nucleus to cytoplasmic sites and appears to be important in mammalian spermatogenesis [177–180]. Although it is expressed in female *N. agilis* specimens too, its expression was significantly increased in males (male-biased). The second of these transcripts, this time female-biased in expression, matches the gene coding for Cytoskeleton-associated protein 5 (CKAP5). This protein is a microtubule polymerase that plays a major role in organizing the spindle pole during mitosis and might also be essential to meiosis and embryonic neural development [175, 176]. Another female-biased transcript that showed positive correlation between abundance and worm size seems homologous to the protein NO VEIN, or Wu: fj29h11. This protein is required for plant embryogenesis, at least in the model species *Arabidopsis thaliana*, but no homolog has been characterized in metazoans before. Finally, two transcripts showed a positive correlation between abundance and worms’ total RNA weight in both sexes and were not significantly sex-biased. The first one is *VARS* and the second one was annotated as *CDC53* or *cul-2*. Both code for core components of multiple cullin-RING-based E3 ubiquitin-protein ligase complexes and can be implicated in various processes such as DNA replication and cell cycle regulation [181, 182]. As discussed earlier, genes with increasing expression as a function of size seem congruent with increasing reproductive activity.

Besides the above-mentioned genes responsible for the production of fibrillar collagen (*COL1A1*, *COL2A1*, *COL11A1*), six annotated transcripts were downregulated in growing worms. One of these was *PCSK2*, which codes for the prohormone convertase 2, involved in the first step in the maturation of many neuroendocrine peptides [183]. Two additional ones were homologous to genes coding for retrotransposon polyproteins, Pol (also male-biased in *N. agilis*) and Ty3 GAG3. In yeasts, the Ty3/Gypsy retrotransposons target RNA Pol III-transcribed genes very precisely, integrating 2–3 bp upstream [184, 185]. Ty3/Gypsy elements get transcribed during meiotic divisions, but their retrotransposition is combated by the cell machinery, which prevents translation of the *GAG3* mRNA, thus maintaining genome integrity [186]. The decline in their expression in *N. agilis* might be associated with a decrease in meiotic activity, or with the onset of germline defense mechanisms downregulating their transcription. A choline transporter gene (*ChT*), in charge of importing the precursor of acetylcholine into neurons, was also found downregulated in larger worms in the present study. As the cholinergic system is involved in the autonomous nervous system and regulates motor functions [187], the pattern might reflect the need for greater mobility in smaller than larger worms. Likewise, the enrichment of actin filament-based process and isoprenoid metabolic process by genes negatively correlated with size in males is congruent with reduced mobility in larger compared to smaller worms. Again, smaller individuals are probably disadvantaged in the competition against larger worms and might need to relocate for a longer time until they find a suitable spot along the host intestine, as outlined above. Finally, two additional male-biased transcripts were upregulated in smaller worms of both sexes; these were homologous to *DDX17* and *PDLIM4*. The first one codes for probable ATP-dependent RNA helicase that might be involved in a variety of processes, linked to chromatin organization, transcriptional regulation and cell differentiation, among others [188, 189]. The second one codes for an actin-associated protein involved in the reorganization of the cytoskeleton, in particular by stimulating actin bundling [190].

Equidirectional relationships between abundance and size in both males and females make these genes good candidate targets for acanthocephalan control in fish aquaculture, with the exception of the Ty3 retrotransposon genes, which do not seem to play a role in the development and reproduction of *N. agilis*. The same way gene silencing by RNA interference (RNAi) is being developed with the hope of making it a sustainable and specific method of pest control in crops [191], RNAi could be used to kill or sterilize acanthocephalan parasites in aquatic farming [192]. Several challenges will need to be tackled along the way. One of them will be to design specific dsRNA fragments that could be taken in by the fish, without being degraded in the digestive system and without affecting it. Extensive testing in lab conditions will be needed, not only to validate targets and delivery methods, but to ensure specificity to *Neoechinorhynchus* parasites and safety for other aquatic organisms.

## Conclusions

Despite their wide distribution and economic relevance of acanthocephalans, molecular data and knowledge on the life history of acanthocephalans are comparably scarce [193]. This led us to investigate the molecular background of acanthocephalan development and reproduction in the fish parasite *N. agilis*. In detail, we reconstructed a draft genome assembly and transcriptome from 74 *N. agilis* specimens, of which 66 were kept for differential expression analysis. These covered males and females of different sizes and maturation stages. The metrics of the current genome and transcriptome assemblies are consistent with the expectation for a member of the Acanthocephala-Rotifera clade (Rotifera, syn. Syndermata). This includes a genome of about 46 Mb harboring about 13,700 coding genes, the exons of which occupy about 31.6% of the genome. Functional annotations of transcripts with male-biased abundances emphasize the particular role of energy metabolism and sperm production in male life. In turn, developmental engagements are more prominently represented in transcripts with a female bias in abundance. The differences in transcriptome profiles reflect the high morphological disparity of male and female worms. Lower competitiveness of males, which are smaller than females, might contribute to the energetic challenges in male life. In contrast, females may overall be more competitive for the energetically preferable sites along the alimentary canal of their definitive hosts, due to their larger size. Not least, 14 annotated transcripts showed equidirectional abundance-size correlations in males and females, which qualifies the coding genes as candidate targets for acanthocephalan control in fish aquaculture.

## Supplementary Information

Supplementary Material 1.

Supplementary Material 2.

## Data Availability

The datasets, transcriptome, and scripts generated during the current study are available in the open-access Zenodo repository 10.5281/zenodo.17199078. RNA raw reads were deposited in GenBank with the BioProject accession number PRJNA1223661. The Whole Genome Shotgun project has been deposited at DDBJ/ENA/GenBank under the accession JBMGUZ000000000. The version described in this paper is version JBMGUZ010000000. The transcriptome of *S. nebaliae*, assembled by Mauer et al. [78], can be found in the Zenodo repository 10.5281/zenodo.17198800, and the transcriptome of *P. laevis*, assembled by Mauer et al. [77], can be found in the Zenodo repository 10.5281/zenodo.17198956. Competing interests. The authors declare that they have no competing interests.
