# Supplementary material for: Transcriptional profiles of the fish parasite Neoechinorhynchus agilis (Acanthocephala) emphasize energetic stress in males and high cell-division activity in females
Source: BMC Genomics. 2025 Dec 9;26:1090. doi: 10.1186/s12864-025-12298-y (PMC12690905; doi:10.1186/s12864-025-12298-y)
Supplement: Supplementary file 2 — Supplementary Material 2. [file 12864_2025_12298_MOESM2_ESM.docx]

Supplementary Material

Transcriptional profiles of the fish parasite *Neoechinorhynchus agilis* (Acanthocephala) emphasize energetic stress in males and high cell-division activity in females.

Camille-Sophie Cozzarolo, Alexandros Vasilikopoulos, Olivier De Thier, Laura Hagemann, Bahram Sayyaf Dezfuli, Karine van Doninck, Holger Herlyn

# Supplementary methods

## Genome assembly with Oxford Nanopore Technology (ONT) reads

In addition to the genome assembly generated with MaSuRCA, we generated a long-read-only genome assembly to determine the assembly with the highest completeness for downstream analyses. The genome assembly completeness might be critical for accurate gene prediction and subsequent differential gene expression analysis. First, we used the same set of filtered ONT reads as described in the main text to assemble a draft genome using the software Flye v. 2.9.1 [1]⁠. We used the option ‘--nano-raw’ for long noisy reads for which Flye automatically applies a correction. We also used the option ‘--no-alt-contigs’ to minimize the amount of uncollapsed haplotypes in the inferred draft genome. Subsequently, we proceeded with removing remaining uncollapsed haplotypes with purge_dups v. 1.2.5 as was done for the hybrid assembly generated with MaSuRCA (see main text) [2]⁠. Polishing of genome assemblies generated with long reads before scaffolding is essential, since assemblies from long noisy reads can contain many errors, potentially interfering with the correct mapping of Hi-C reads [3]⁠. Such errors may be short indels, GC bias, and homopolymers [3]⁠. We therefore proceeded by polishing the assembled genome that resulted from Flye analysis by making use of the high-accuracy Illumina reads. Polishing of the long-read assembly was performed with the software Nextpolish v. 1.4.0 [4]⁠.

We used BUSCO v. 5.4.3 to compare the completeness of these genome assemblies with the assembly inferred with the hybrid mode from MaSuRCA [4]⁠. These comparisons were made by using the options ‘--augustus --augustus_species schistosoma’ in the BUSCO analysis. We selected ‘schistosoma’ as the taxon for training Augustus within BUSCO because it is more closely related to *Neoechinorhynchus agilis* than the default species *Drosophila melanogaster* [5]. The statistics of the final scaffolded assembly were based on the same version of BUSCO but without using the ‘-–augustus’ option. This approach uses MetaEuk instead of Augustus for gene finding and was selected since it produced the highest completeness scores for the final genome assembly. We also used the high-accuracy Illumina reads as a reference to compare k-mer completeness of the assembled genomes before scaffolding with Hi-C data. For this task, we used the K-mer Analysis toolkit v. 2.4.2 [6]⁠

## Verification of sex identification

In order to check the accuracy of sex identification by visual inspection, we used the list of annotated genes that showed sex-biased gene expression in *Pomphorhynchus laevis* presented in Table 2 of [7] and selected the *N. agilis* transcripts that received the same annotation. This resulted in a matrix of variance-stabilized gene counts containing the 229 transcripts that were annotated with one of these homologs. These transcripts are thereafter referred to as "sex markers". We then produced a heatmap of sample-to-sample distances (supplementary Fig. S6), which revealed two points: 1) as suspected, five samples visually assigned females clustered with males and two samples visually assigned males clustered with females, and 2) six samples (five visually assigned males and one visually assigned female) showed expression patterns dissimilar to worms of both sexes. When performing hierarchical clustering using K=2 and the "complete" method, these six datapoints clustered with the females; when using the "average" method, they clustered with the males. When using K=3, with both methods, they formed their own cluster.

To further investigate this matter, we also conducted a PCA based on the expression of these "sex markers". Again, the presumed misidentified samples clustered with samples of the other sex (supplementary Fig. S7). The outliers loaded between both male and female clusters on PC1 (explaining 57% of variance) and more negatively than the others on PC2 (explaining 12% of variance). We excluded the seven likely misassigned worms from downstream analyses, thus focusing on the remaining 36 male and 30 female individuals in expressional analyses. Sample clustering based on the expression of "sex markers" revealed six additional datapoints with intermediate or diverging expression profiles. We kept these in the dataset with their visually assigned sex, since an intermediate position does not rule out correct sexing.

## References

1. Kolmogorov M, Yuan J, Lin Y, Pevzner PA. Assembly of long, error-prone reads using repeat graphs. Nature Biotechnology. 2019;37:540–6. https://doi.org/ 10.1038/s41587-019-0072-8

2. Guan D, McCarthy SA, Wood J, Howe K, Wang Y, Durbin R. Identifying and removing haplotypic duplication in primary genome assemblies. Bioinformatics. 2020;36:2896–8. https://doi.org/ 10.1093/bioinformatics/btaa025

3. Guiglielmoni N, Rivera-Vicéns R, Koszul R, Flot J-F. A deep dive into genome assemblies of non-vertebrate animals. Peer Community Journal. 2022;2. https://doi.org/10.24072/pcjournal.128

4. Hu J, Fan J, Sun Z, Liu S. NextPolish: A fast and efficient genome polishing tool for long-read assembly. Bioinformatics. 2020;36:2253–5. https://doi.org/10.1093/bioinformatics/btz891

5. Struck TH, Wey-Fabrizius AR, Golombek A, Hering L, Weigert A, Bleidorn C, Klebow S, Iakovenko N, Hausdorf B, Petersen M, Kück P, Herlyn H, Hankeln T. Platyzoan paraphyly based on phylogenomic data supports a noncoelomate ancestry of Spiralia. Molecular Biology and Evolution. 2014;31:1833–49. https://doi.org/10.1093/molbev/msu143

6. Mapleson D, Accinelli GG, Kettleborough G, Wright J, Clavijo BJ. KAT: A K-mer analysis toolkit to quality control NGS datasets and genome assemblies. Bioinformatics. 2017;33:574–6. https://doi.org/10.1093/bioinformatics/btw663

7. Schmidt H, Mauer K, Hankeln T, Herlyn H. Host-dependent impairment of parasite development and reproduction in the acanthocephalan model. Cell & Bioscience. 2022;12:75. https://doi.org/10.1186/s13578-022-00818-2.

# Supplementary Tables

**Table S1 Completeness and contiguity statistics of the generated genome assemblies pre-caffolding and their comparison to the final genome assembly.** Note that for comparing the unscaffolded assemblies, the –augustus option was used in BUSCO (see supplementary methods). The primary assemblies that resulted from MaSuRCA and Flye were processed with purged_dups to remove uncollapsed haplotypes. The long-read-only assembly was then polished with the use of short Illumina reads. The final scaffolded assembly refers to the assembly after manual curation, and after removing short contigs and potential contaminants. Note that the MaSuRCA assembly overall showed higher completeness than the long-read-only assembly (see also k-mer spectra copy number plots).

| **Assembly** | **k-mer completeness (%)** | **BUSCO complete (%) - Metazoa odb10 (with Augustus)** | **BUSCO complete (%) - Eukaryota odb10 (with Augustus)** | **BUSCO complete (%) - Metazoa odb10 (with MetaEuk)** | **BUSCO complete (%) - Eukaryota odb10 (with MetaEuk)** | **Genome assembly size (bp)** | **N50**  **(bp)** |
| --- | --- | --- | --- | --- | --- | --- | --- |
| Final scaffolded assembly | 96.35 | 38.5 | 38.8 | 47.1 | 58.4 | 46,219,100 | 16,375,937 |
| **MaSuRCA** genome assembly + purge_dups | 96.51 | 38.0 | 40.4 | Not performed | Not performed | 46,491,529 | 482,219 |
| **Flye** ONT-only + purge_dups | 93.47 | 37.5 | 38.1 | Not performed | Not performed | 46,686,715 | 269,451 |
| **Flye** ONT-only + purge_dups + polished | 93.88 | 37.8 | 38.1 | Not performed | Not performed | 46,676,564 | 269,443 |

**Table S2** Metrics of the de novo assembled *N. agilis* genome as outputted by QUAST.

| **Metric** | **Value** |
| --- | --- |
| Number of contigs (>= 0 bp) | 102 |
| Number of contigs (>= 1000 bp) | 102 |
| Number of contigs (>= 5000 bp) | 102 |
| Number of contigs (>= 10,000 bp) | 83 |
| Number of contigs (>= 25,000 bp) | 37 |
| Number of contigs (>= 50,000 bp) | 14 |
| Total length (>= 0 bp) | 46,219,100 |
| Total length (>= 1000 bp) | 46,219,100 |
| Total length (>= 5000 bp) | 46,219,100 |
| Total length (>= 10,000 bp) | 46,074,715 |
| Total length (>= 25,000 bp) | 45,287,003 |
| Total length (>= 50,000 bp) | 44,463,112 |
| Number of contigs | 102 |
| Largest contig | 19,375,080 |
| Total length | 46,219,100 |
| GC (%) | 38.81 |
| N50 | 16,375,937 |
| N90 | 7816151 |
| auN | 15,248,791.9 |
| L50 | 2 |
| L90 | 3 |
| Number of N's per 100 kbp | 0.9 |

**Other tables: see in supplementary_tables.xlsx**

# Supplementary Figures


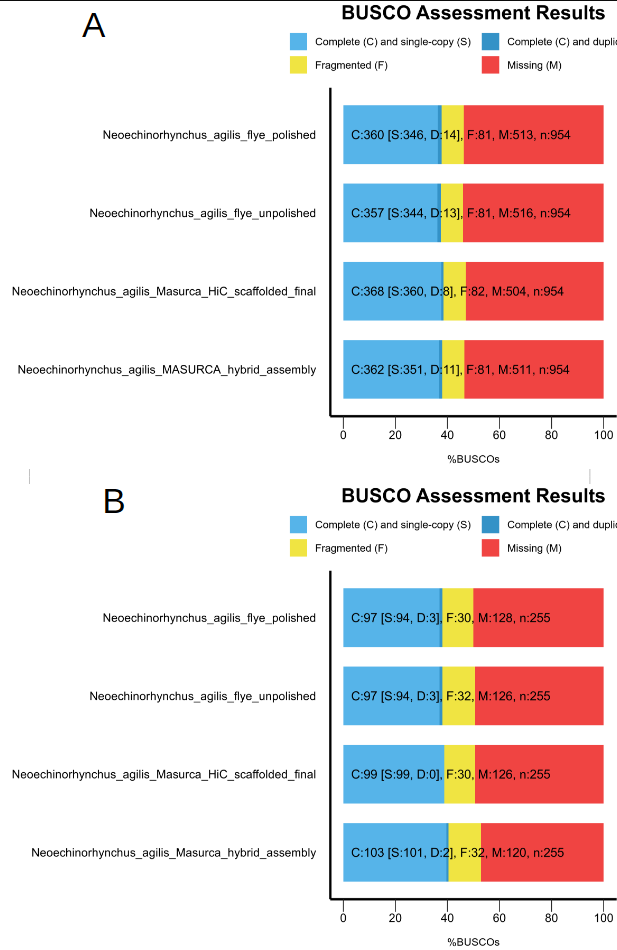


**Figure S1 Assessment of genome completeness of alternative *N. agilis* genome assemblies.** Plots generated using the BUSCO software that show comparisons of Universal single-copy ortholog completeness across the genome assemblies generated. A) Comparison of completeness using the Matazoa odb10 BUSCO Database as a reference, B) Comparison of completeness using the Eukaryota odb10 Database as a reference.


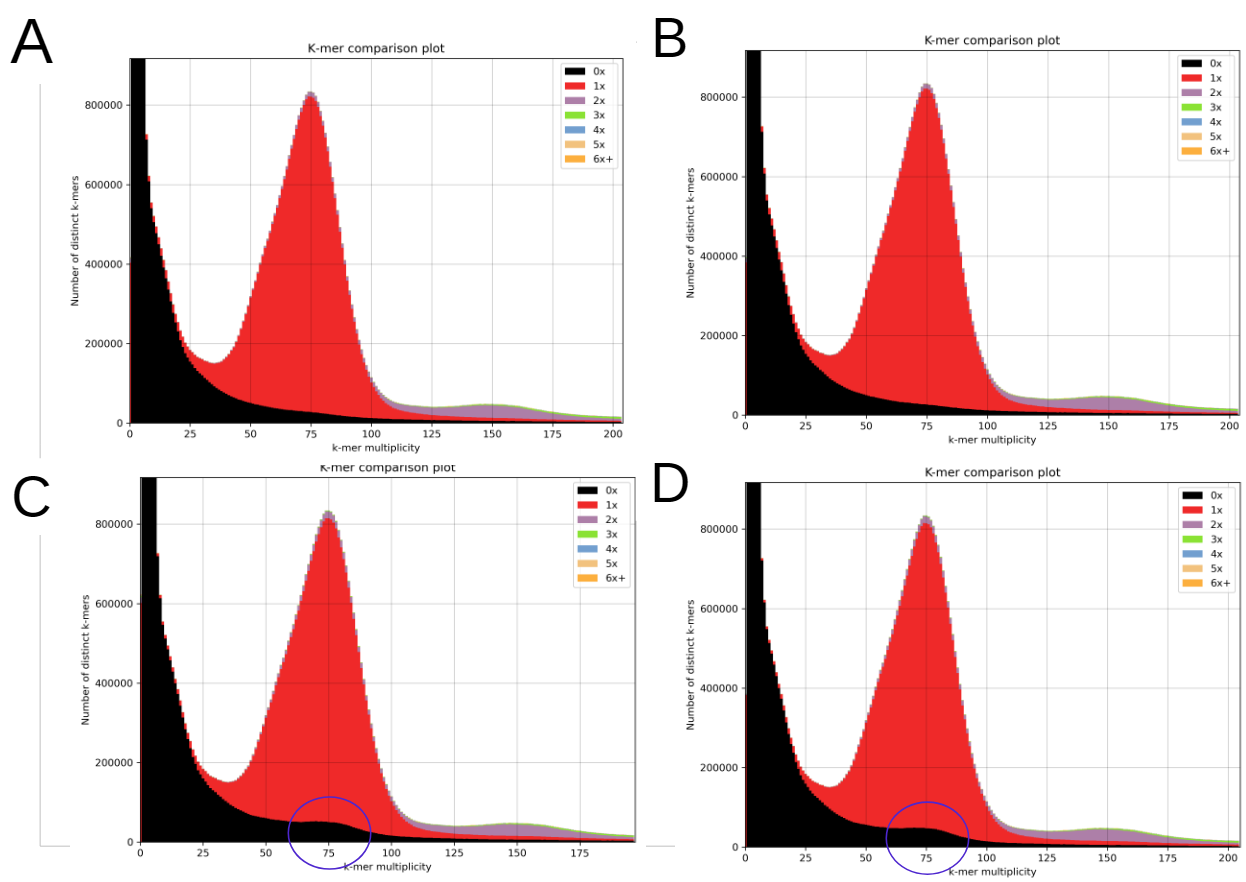


**Figure S2 k-mer spectra for alternative *N. agilis* genome assemblies.** The k-mer spectrum of Illumina reads was used as a reference. Missing k-mers from the genome assembly are shown in black. Blue circles show areas of potentially missing k-mers at the region of homozygous content for the genome assemblies generated with Flye. This suggests that some Illumina genome content is missing from these assemblies (see also supplementary Table S1). Since we did not detect profound evidence of contamination in the MaSuRCA genome assembly, we consider these missing k-mers do not originate from contamination. A) Final scaffolded genome assembly, B) MaSuRCA hybrid genome assembly, C) Flye genome assembly, D) Flye genome assembly after polishing.


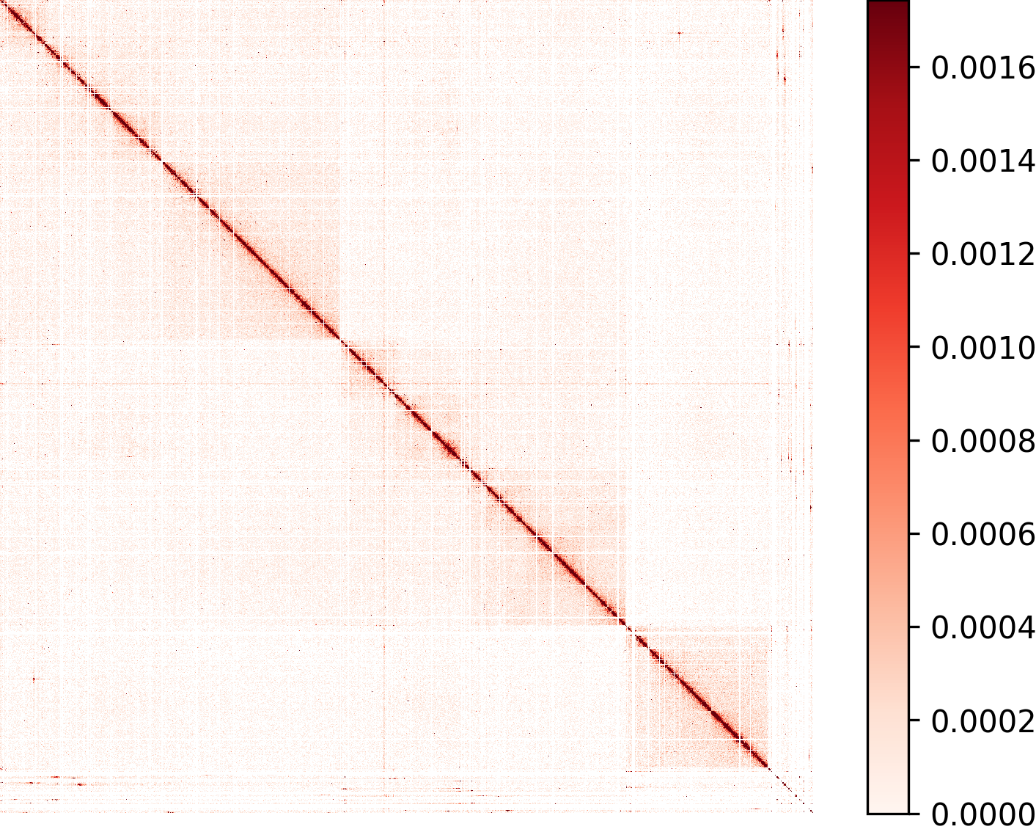


**Figure S3 Hi-C heatmap for the *N. agilis* genome.** Three scaffolds in the haploid genome are visually distinguishable by darker red, reflecting distinct chromosomal contact domains.


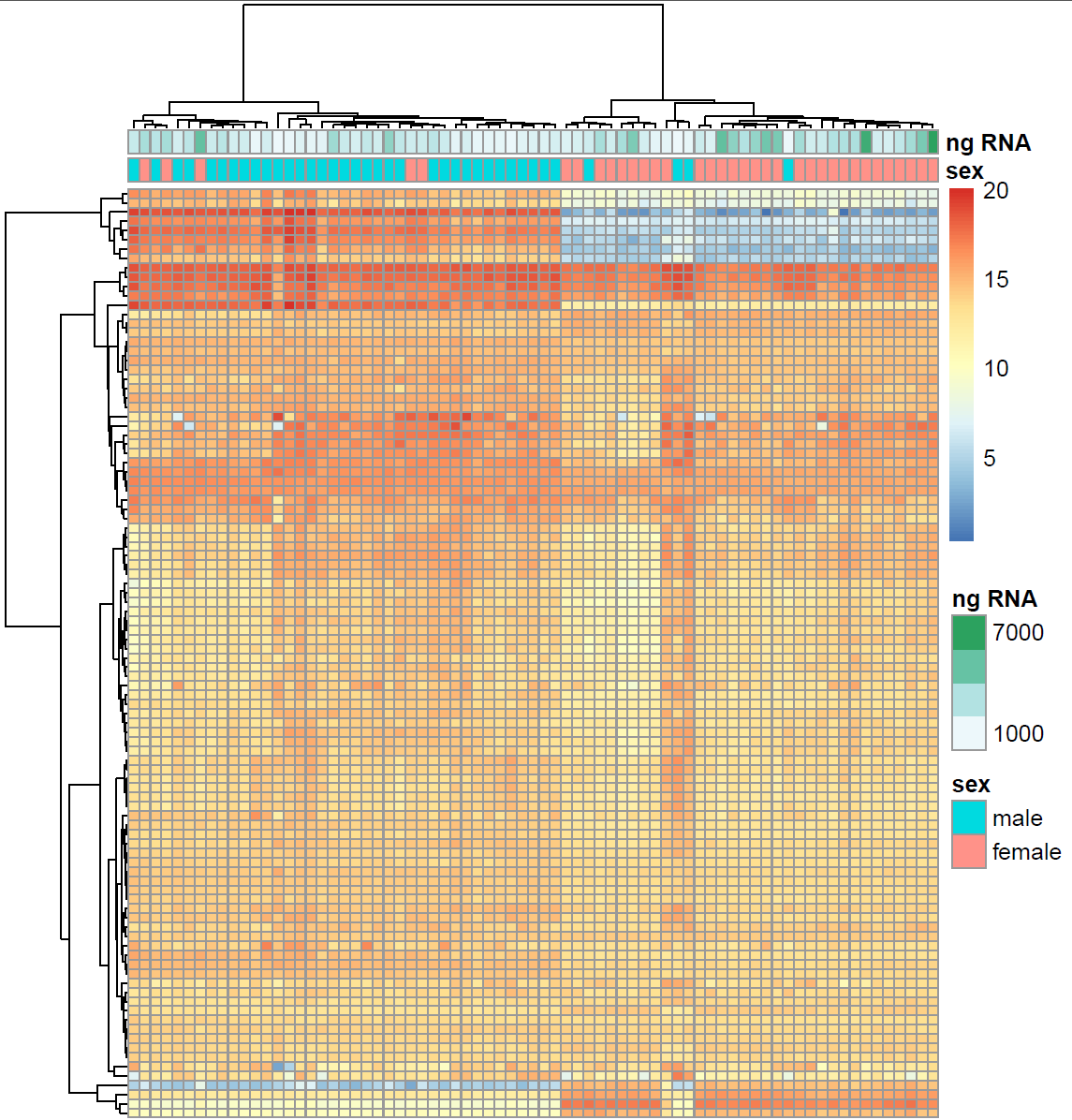


**Figure S4 Heatmap of 74 *N. agilis* transcriptome profiles based on variance-stabilized abundances.** The heatmap shows the 100 transcripts with the highest mean of variance-stabilized counts. The lowermost annotation row indicates the sex: orange for females and blue for males. The uppermost annotation row shows total RNA weight as isolated from single worms (light to dark green: small to large RNA amount).


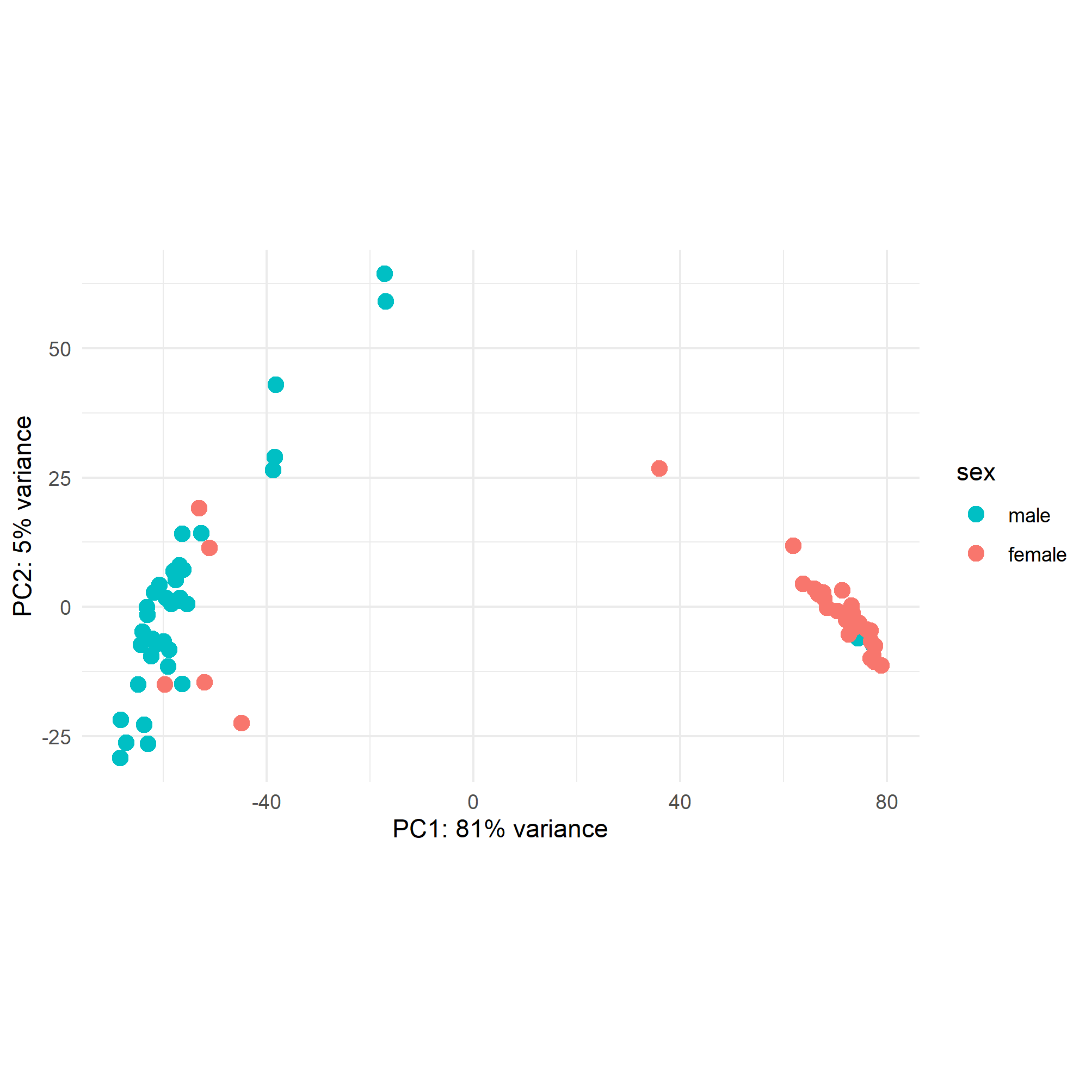


**Figure S5 Principal Component (PC) Analysis of 74 *N. agilis* transcriptome profiles based on variance-stabilized abundances.**


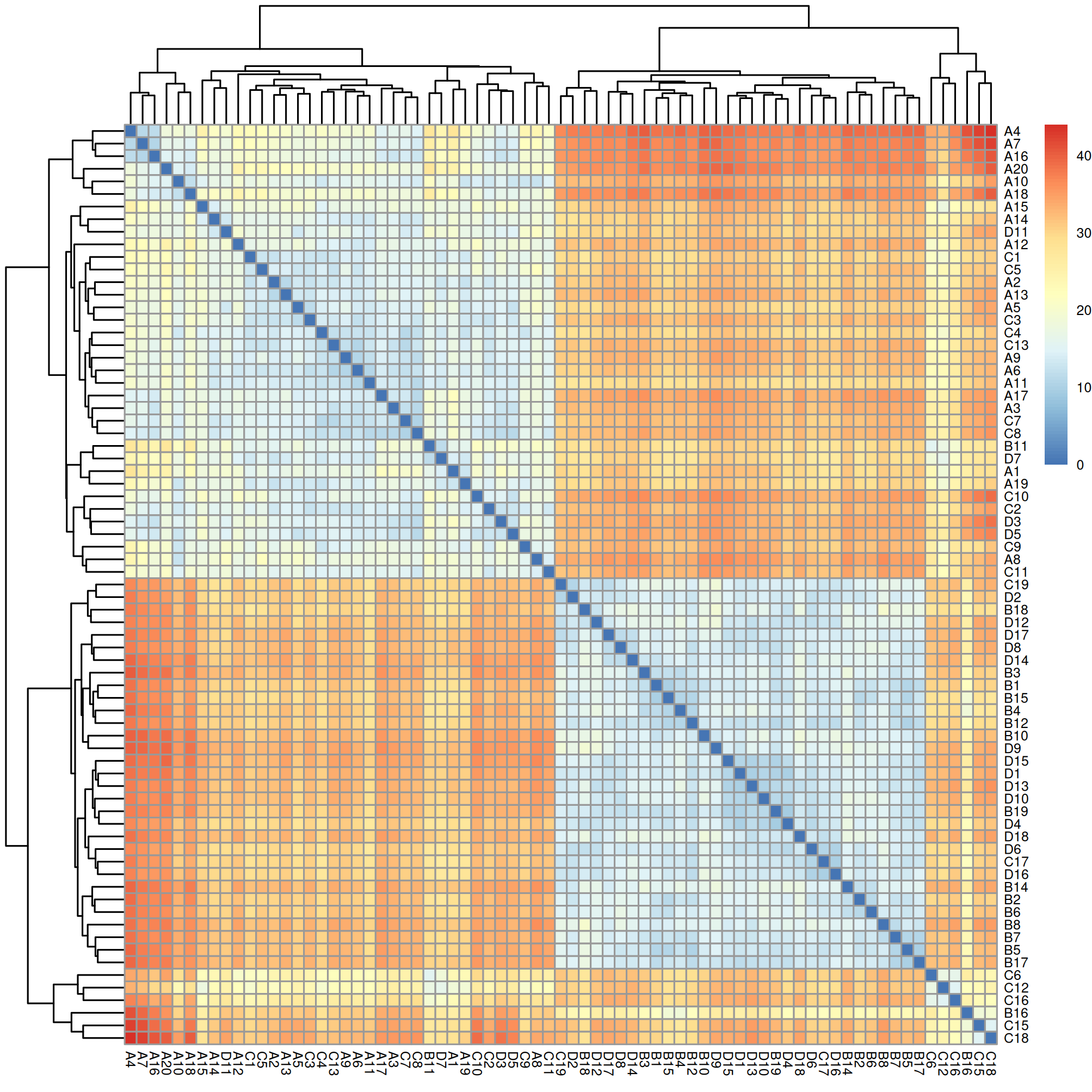


**Figure S6 Sample-to-sample heatmap based on the variance-stabilized counts of *N. agilis* genes homologous to sex-biased genes in *P. laevis*.**

**
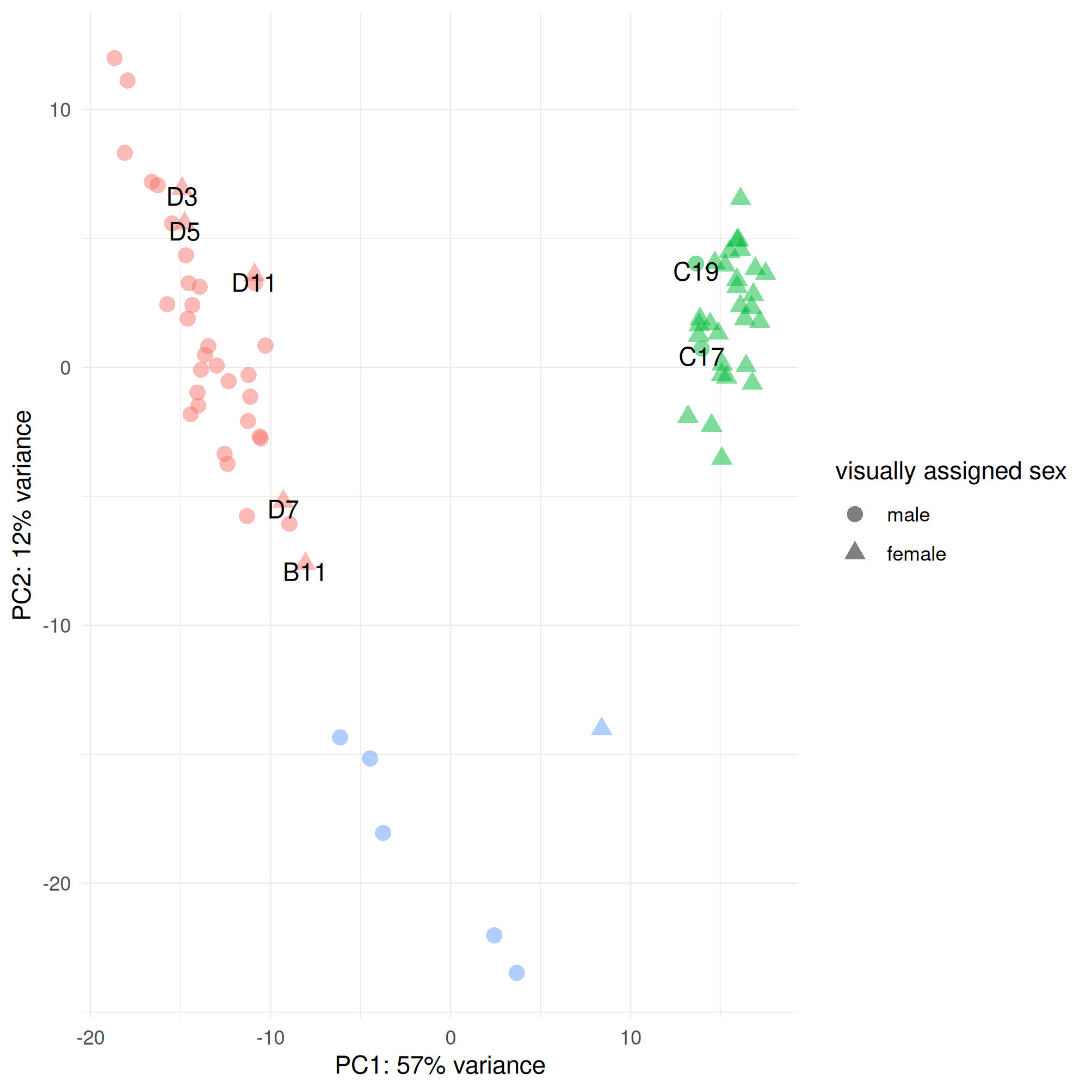
**

**Figure S7 Principal Component (PC) Analysis of 74 *N. agilis* transcriptome profiles based on variance-stabilized abundances of genes sex-biased in *P. laevis*.** Triangles and circles represent females and males, respectively. The colors indicate the clusters to which the samples were attributed by hierarchical clustering when using k=3. The samples annotated with their ID are those that cluster with the samples that were visually assigned to the other sex and were removed from the dataset before DEA. There is no deeper reason for some female specimens being labelled B and others D.


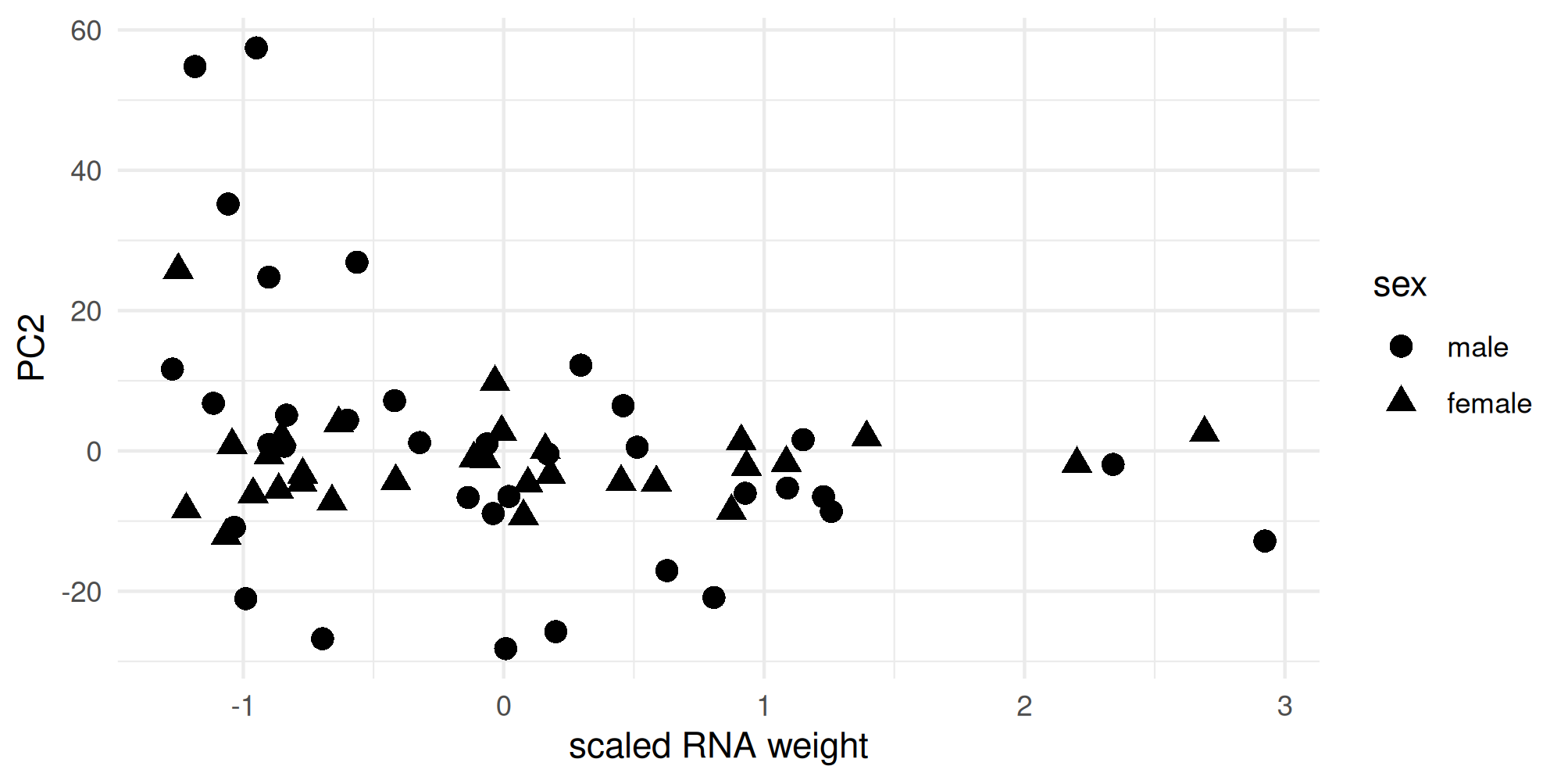


**Figure S8 Association between the first three Princpal Component (PC) axes and RNA content.** The Breusch-Pagan test indicated heteroscedasticity in the association between PC2 and scaled RNA content, confirming the highest variance in expression profiles of smaller worms.


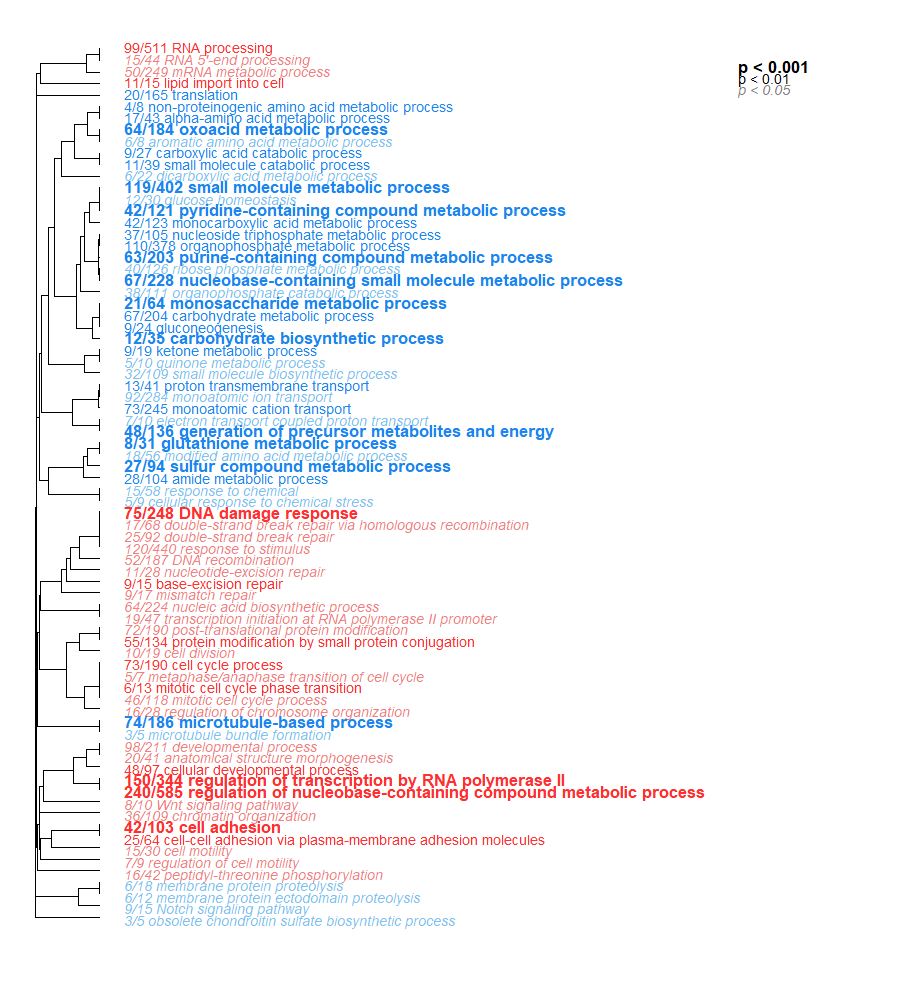


**Figure S9 Rank-based Gene Ontology (GO) enrichment analysis for Biological Processes in females compared to males.** The ranking is based on log2(fold change). Font types represent adjusted significance levels (bold: p-value < 0.001; normal: p < 0.01; italics: p < 0) from the Mann-Whitney U test, corrected with Benjamini-Hochberg (1995: see References in main text) false discovery rate. Colors represent the rank sign: red terms have a positive mean rank, meaning the genes annotated with them are on average upregulated in females. Blue represents male-biased terms. The fractions preceding the terms represent the number of "good candidates" relative to the total number of genes belonging to this category. "Good candidates" are genes with an absolute log2(fold change) > 1.


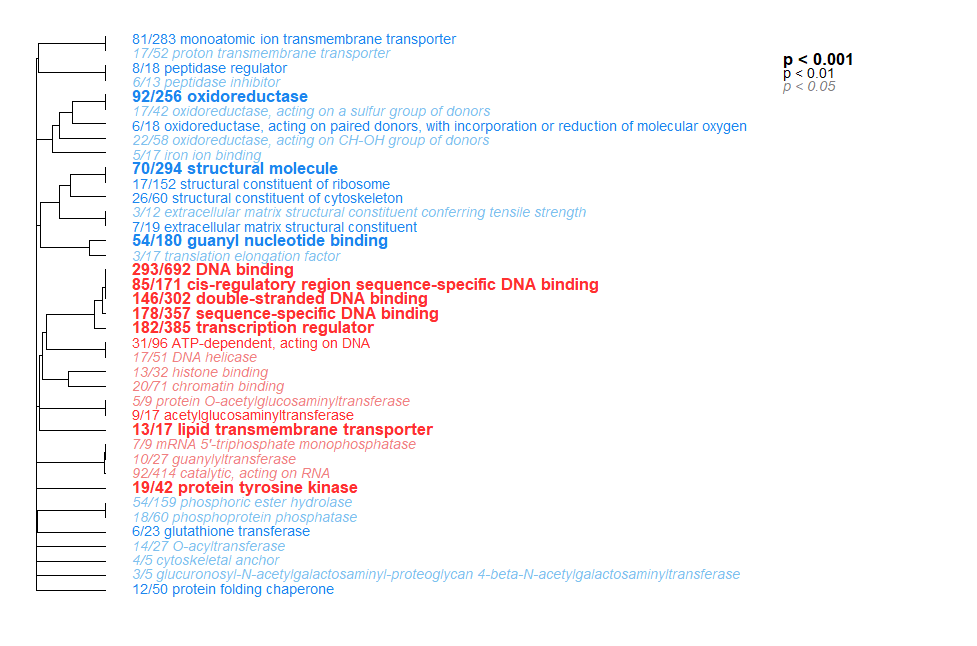


**Figure S10 Rank-based Gene Ontology (GO) enrichment analysis for Molecular Function in females compared to males.** The ranking is based on log2(fold change). Font types represent adjusted significance levels (bold: p-value < 0.01; normal: p < 0.05; italics: p < 0.1) from the Mann-Whitney U test, corrected with Benjamini-Hochberg (1995: see References in main text) false discovery rate. Colors represent the rank sign: red terms have a positive mean rank, meaning the genes annotated with them are on average upregulated in females. Blue represents male-biased terms. The fractions preceding the terms represent the number of "good candidates" relative to the total number of genes belonging to this category. "Good candidates" are genes with an absolute log2(fold change) > 1.

| A  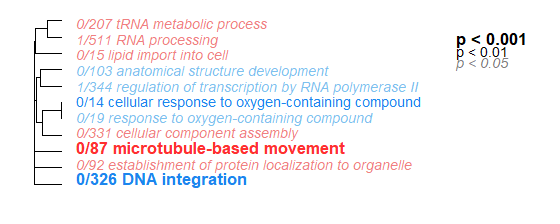 | B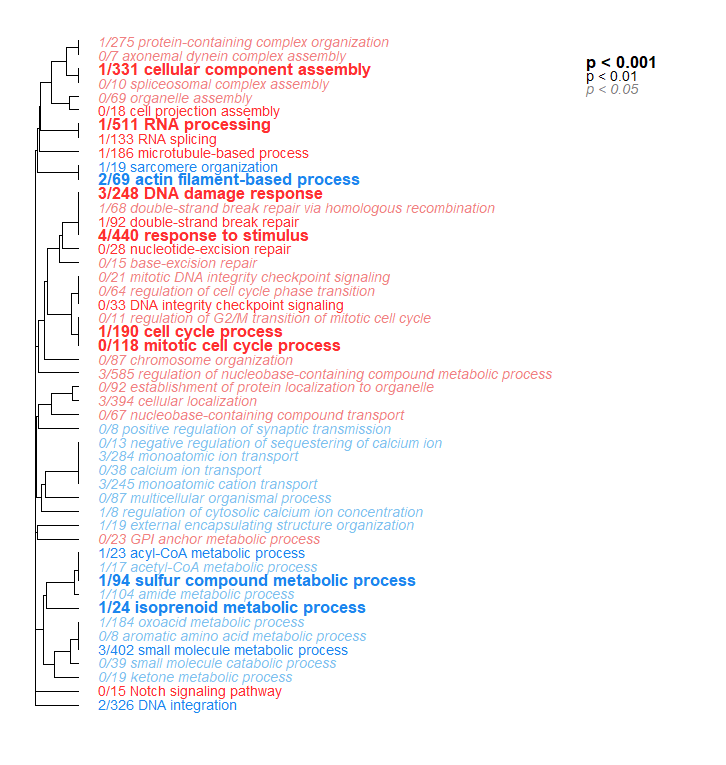 |
| --- | --- |
| C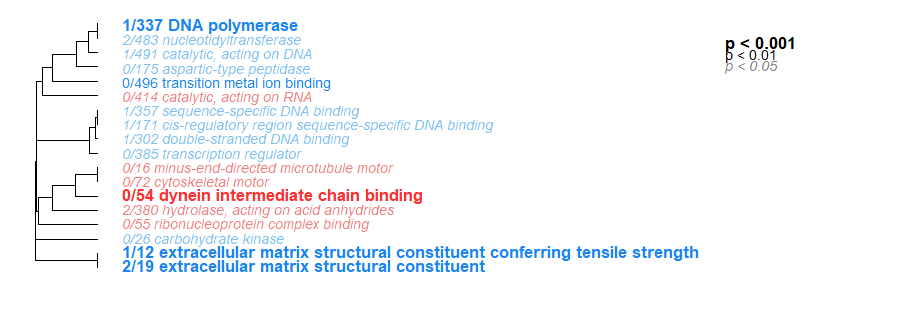 | D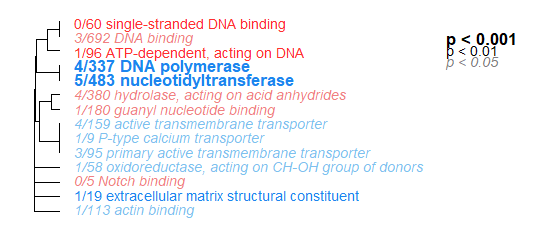 |

**Figure S11 Rank-based Gene Ontology (GO) enrichment analysis (previous page)**. Colors represent the rank sign: red terms have a positive mean rank, meaning the genes annotated with them show a positive association between expression and worm size. Blue represents terms enriched in genes negatively correlated with size. The ranking is based on log2(fold change). Font types represent adjusted significance levels (bold: p-value < 0.01; normal: p < 0.05; italics: p < 0.1) from the Mann-Whitney U test, corrected with Benjamini-Hochberg (1995: see References in main text) false discovery rate. A) Biological Process, in females; B) Biological Process, in males; C) Molecular Function, in females; D) Molecular Function, in males. The fractions preceding the terms represent the number of "good candidates" relative to the total number of genes belonging to this category. "Good candidates" are genes with an absolute log2(fold change) > 1.

**
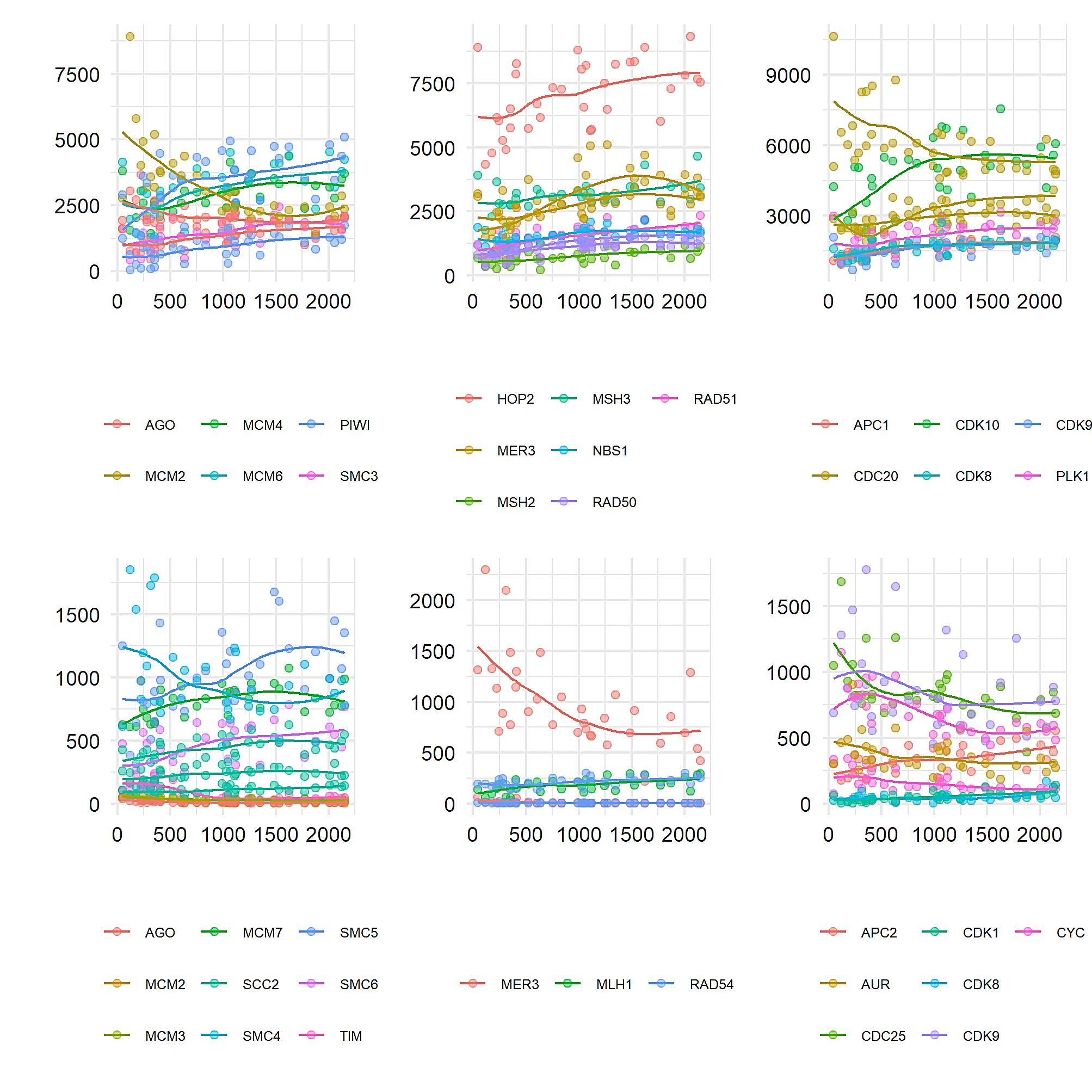
Figure S12 Expression of meiosis-related genes.** Variance-stabilized counts are shown in relation to male whole-body RNA weight. The genes presented here were gathered in an inventory by Hanson et al. [111] because they play a role in meiosis in model organisms. They were further categorized as involved in meiosis entry, DNA replication, and chromosome structure (left), meiotic recombination (middle) and meiosis progression (right). Only transcripts that were significantly (p < 0.05) correlated with total RNA weight are shown. The curves were plotted using geom_smooth to visualize trends and do not represent the DESeq2 model coefficients. Two outliers (worms that yielded more than 3000 ng of RNA) were removed to examine whether they were driving the observed trends. Genes with average variance-stabilized counts > 1000 are shown on the top, and those with < 1000 are shown in the bottom, for the sake of readability.
